# Head-to-head comparisons of *Toxoplasma gondii* and its near relative *Hammondia hammondi* reveal dramatic differences in the host response and effectors with species-specific functions

**DOI:** 10.1371/journal.ppat.1008528

**Published:** 2020-06-23

**Authors:** Zhee Sheen Wong, Sarah L. Sokol-Borrelli, Philip Olias, J. P. Dubey, Jon P. Boyle

**Affiliations:** 1 Department of Biological Sciences, Dietrich School of Arts and Sciences, University of Pittsburgh, Pittsburgh, Pennsylvania, United States of America; 2 University of Bern, Bern, Switzerland; 3 Animal Parasitic Diseases Laboratory, Beltsville Agricultural Research Center, Agricultural Research Service, U.S. Department of Agriculture, Beltsville, Maryland, United States of America; University of Wisconsin Medical School, UNITED STATES

## Abstract

*Toxoplasma gondii* and *Hammondia hammondi* are closely-related coccidian intracellular parasites that differ in their ability to cause disease in animal and (likely) humans. The role of the host response in these phenotypic differences is not known and to address this we performed a transcriptomic analysis of a monocyte cell line (THP-1) infected with these two parasite species. The pathways altered by infection were shared between species ~95% the time, but the magnitude of the host response to *H*. *hammondi* was significantly higher compared to *T*. *gondii*. Accompanying this divergent host response was an equally divergent impact on the cell cycle of the host cell. In contrast to *T*. *gondii*, *H*. *hammondi* infection induces cell cycle arrest via pathways linked to DNA-damage responses and cellular senescence and robust secretion of multiple chemokines that are known to be a part of the senescence associated secretory phenotype (SASP). Remarkably, prior *T*. *gondii* infection or treatment with *T*. *gondii*-conditioned media suppressed responses to *H*. *hammondi* infection, and promoted the replication of *H*. *hammondi* in recipient cells. Suppression of inflammatory responses to *H*. *hammondi* was found to be mediated by the *T*. *gondii* effector IST, and this finding was consistent with reduced functionality of the *H*. *hammondi* IST ortholog compared to its *T*. *gondii* counterpart. Taken together our data suggest that *T*. *gondii* manipulation of the host cell is capable of suppressing previously unknown stress and/or DNA-damage induced responses that occur during infection with *H*. *hammondi*, and that one important impact of this *T*. *gondii* mediated suppression is to promote parasite replication.

## Author summary

Humans can be infected with *Toxoplasma gondii* from undercooked meat or exposure to feces from infected cats, and this parasite can cause lethal disease in those with HIV/AIDS, immunosuppressed organ transplant patients, and in the developing fetus. *T*. *gondii* is a global parasite of all warm-blooded animals and has infected over a billion people worldwide, making it one of the more successful parasites on earth. To better understand why *T*. *gondii* is such a successful parasite, we have taken a comparative approach to investigate the role of the host response in determining infection outcome to *T*. *gondii* and its nearest extant relative, *Hammondia hammondi*. These parasite species are closely related, sharing nearly all of their genes, but *H*. *hammondi* is not known to causes disease in humans and is highly avirulent in all animal models studied to date. Here we performed the first head-to-head comparison of the host response to these two parasite species and found a) that *H*. *hammondi*-infected cells exhibited a more robust host response than those infected with *T*. *gondii* and b) that *T*. *gondii* can suppress host responses to *H*. *hammondi*. These studies not only implicate the acute host response as a mechanism for increased control of *H*. *hammondi in vivo*, but also reveal previously unknown immune suppression mechanisms in *T*. *gondii*.

## Introduction

A successful infection in *T*. *gondii* results in rapid replication of infecting parasites as tachyzoites, dissemination to a variety of tissues, followed by the clearance of actively replicating stages and encystment of bradyzoite stages in muscle and neuronal tissues. Therefore the ability of *T*. *gondii* to suppress host responses are important to promote its rapid growth and dissemination during the acute phase such that a sufficient number of parasites can ultimately convert to the cyst stage and therefore be transmitted to the next host.

A critical feature of *T*. *gondii* infection is the ability to alter fundamental biological processes of the host cell. *T*. *gondii* causing changes in multiple pathways including those responsible for the regulation of metabolism [1] and innate immune signaling [2], and many of these are known to be important for parasite replication and dissemination during the acute phase. Many of the transcriptional changes that occur during *T*. *gondii* infection depend on the secretion of specific effectors directly into the host cell [1, 3, 4], and *T*. *gondii* is likely to harbor hundreds of such effectors [5, 6]. Rhoptry-derived proteins such as ROP16 regulate host transcription factors like STAT3 [7] and STAT6 [8], while dense granule proteins GRA15 [9], GRA16 [10] and GRA24 [11] modulate host NF-κB, p53 and p38 MAP kinase host pathways, respectively. GRA18 modulates the Wnt signaling pathway in mouse cells, owing to its ability to stabilize the transcription factor β-catenin [12], and GRA25 induces CCL2 production by human foreskin fibroblasts [13]. The parasite effector TgIST represses the interferon (IFN)-γ response by recruiting the Mi-2/NuRD repressor complex and subsequently blocking STAT1-related IFNγ-stimulated transcription [14, 15]. For the dense granule-derived effectors, many require the presence of a protein complex on the parasitophorous vacuole membrane (PVM) consisting of at least three proteins named MYR1, 2 and 3 [16, 17]. This complex has also been shown recently to be critical for the induction of the chemokine CCL22 in human placental cells [18]. Importantly *T*. *gondii* has no impact on CCL22 production by human foreskin fibroblasts (HFFs) [18], further demonstrating that responses to *T*. *gondii* infection can vary dramatically across cell types. Taken together it is clear that multiple means of modulating the host cell exist in *T*. *gondii*.

An unanswered question is how important these host manipulations are in determining the many unique life cycle characteristics of *T*. *gondii* compared to its near Apicomplexan relatives, including the lack of an obligately heteroxenous life cycle and the ability to infect an uncharacteristically large number of intermediate hosts. As a means to address this question we have turned to the nearest extant relative of *T*. *gondii*, *Hammondia hammondi* as a parasite lineage that is naturally deficient in the aforementioned life cycle features. *H*. *hammondi* was first discovered in the feces of a cat in Iowa, USA in 1975 [19], and while morphologically indistinguishable from *T*. *gondii*, *H*. *hammondi* has a comparatively restricted natural intermediate host range (being incapable of infecting birds) [20, 21]. Prior work shows that *H*. *hammondi* shares >95% of its ~8000 genes in near perfect synteny with *T*. *gondii* and expresses functional orthologs of key *T*. *gondii* effectors such as ROP18 and ROP5 [22, 23]. This is important from an epidemiological and evolutionary perspective, as *H*. *hammondi* is not known to cause clinical disease in any naturally infected intermediate (human and other animals) or definitive host [20]. In addition, in tissue culture *H*. *hammondi* replicates for a short period of time and then enters into a unique terminally differentiated bradyzoite state that is a) unable to be subcultured *in vitro*, b) incapable of infecting an intermediate mouse host and c) only capable of infecting a feline host [20, 23]. Paradoxically, despite this natural bradyzoite developmental program and expression of canonical bradyzoite genes during tachyzoite-like replication [23, 24], *H*. *hammondi* cannot be induced to form bradyzoites using stresses like high pH medium that robustly induce cyst formation in *T*. *gondii* [23]. While it is not yet known if *H*. *hammondi* is capable of infecting humans, and there are no tests as yet capable of distinguishing these closely-related species serologically [25], *H*. *hammondi* and *T*. *gondii* oocysts are known to co-circulate, suggesting that humans are likely to encounter this parasite [26].

Since multiple *T*. *gondii* effectors modulate host transcriptional regulation, and to date many of these have been found to be functionally conserved between *T*. *gondii* and *H*. *hammondi* [22, 24], we sought to test the hypothesis that differences in the response of the host cell to *T*. *gondii* and *H*. *hammondi* could be linked to phenotypic differences between these species, including replication rate, life cycle flexibility and pathogenesis. To do this we performed the first thorough analysis of the cell-autonomous host response to *H*. *hammondi* and compared it to *T*. *gondii*. We found that the majority of infection-induced changes in the host cell were conserved between these two species, but also that the magnitude of these changes in *H*. *hammondi*-infected cells was much larger (often by many-fold) than in *T*. *gondii*-infected cells. We confirmed that this effect is manifested at the protein level for a subset of chemokines, and that a similar effect could be observed *in vivo* during mouse infections. In addition to these qualitatively shared pathways, we also identified a small, but important, subset of host cell pathways that were altered during *T*. *gondii* infection but were either unaltered or even inverted in *H*. *hammondi*-infected cells. Some of these transcriptional responses indicated that *H*. *hammondi*-infected cells were in a distinct cell cycle state compared to *T*. *gondii*. Remarkably we also found that prior infection with *T*. *gondii* suppressed host responses to *H*. *hammondi* and that this suppressive effect was partially dependent on the *T*. *gondii* effector IST [14, 15] and that the molecular mechanism for this difference was specific polymorphisms in the TgIST and HhIST coding sequence. Finally we found that prior exposure of non-immune cells to media conditioned with *T*. *gondii*-infected THP-1 cells improved growth conditions for *H*. *hammondi*, linking observed differences in the host response to affecting parasite replication rate in neighboring cells. Taken together these data indicate a high level of conservation of host responses to *T*. *gondii* and *H*. *hammondi*, but extensive divergence in their abilities to target specific host cellular pathways that can be linked to polymorphisms in secreted effectors.

## Results

### *H*. *hammondi* sporozoites induce significantly more potent gene expression changes in THP-1 cells as compared to *T*. *gondii*

To compare the impact of *T*. *gondii* and *H*. *hammondi* infection on the transcriptional host response during infection, we performed RNA-sequencing (seq) on THP-1 cells (a monocyte cell line [27]) infected with representatives of three major lineages of *T*. *gondii* and two *H*. *hammondi* isolates. In all cases (unless specified), we used parasites that were freshly excysted from oocysts and then grown for 24 hours in HFFs prior to being quantified and used to infect THP-1 cells in suspension.

**Fig 1:**
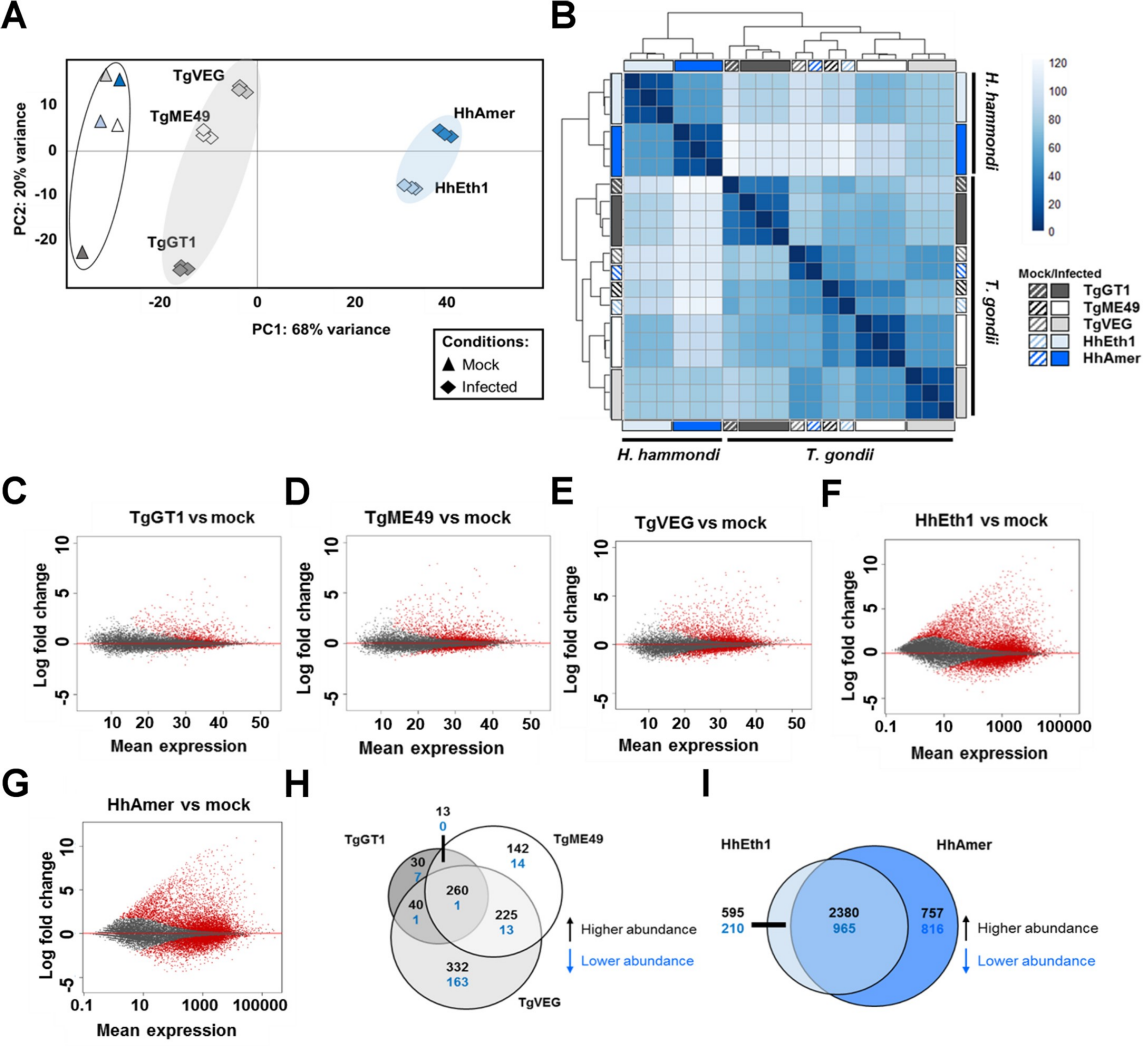
Unique transcriptome profiles of THP-1 cells infected with parasites where *H*. *hammondi* induced higher numbers of differentially expressed genes in THP-1 cells compared to *T*. *gondii*. (A) Principal components (PC) 1 and 2 of THP-1 cells infected (♦) or mock infected (▲) with *T*. *gondii* (TgGT1, Dark grey; TgME49, white; TgVEG, light grey) or *H*. *hammondi* (HhEth1, light blue; HhAmer, dark blue). THP-1 cells infected with *T*. *gondii* clustered together (light grey shading) while THP-1 cells infected with *H*. *hammondi* clustered together along PC1 (light blue shading). *T*. *gondii* and *H*. *hammondi* mock-infected cells (▲) also separated from the parasite-infected cells along PC2. (B) Heatmap showing Euclidean distance clustering between mock (striped boxes) and infected (solid filled boxes) THP-1 cell samples (log_2_-transformed data). (C-G) MA-plots of gene expression in THP-1 cells infected with *T*. *gondii* (Tg) or *H*. *hammondi* (Hh). Red dots (●) represent genes of different abundance in infected THP-1 cells as compared to mock-infected THP-1 cells (alpha = 0.01). (H-I) Venn diagrams of genes in THP-1 cells infected with *T*. *gondii* (H) or *H*. *hammondi* (I; *p*_*adj*_ < 0.01, log_2_ fold-change ≥ 1 or ≤ -1).

We first used principal component analysis (PCA) to look broadly at differences and similarities in the host transcriptome across strains and species [28]. According to this analysis the first PC (PC1, 68% variance) indicated a clear separation between parasite species, while the second (PC2, 20% variance) encapsulated differences between strains of each species and/or experiments that were performed together. These data indicate that THP-1 cells infected with all three *T*. *gondii* types (TgGT1, TgME49 and TgVEG) are more similar to each other than to THP-1 cells infected with either strain of *H*. *hammondi* (HhEth1 and HhAmer; **Fig 1A**). Infection status (Mock vs. Infected) also was distributed across PC1, in that mock-infected cells clustered together within PC1 regardless of strain or species (**Fig 1A**). Importantly the two *H*. *hammondi* strains were much further separated from all mock-infected samples along this PC, suggesting 1) a distinct response by the host cells to this species and/or 2) more potent induction of transcriptional changes by the parasite. PC2 showed a clear separation between TgGT1-infected cells and all other samples, including *T*. *gondii* and *H*. *hammondi*. While it was clear from PCA and other analyses (below) that TgGT1 infection induced extensive changes in transcript abundance compared to mock-infection (Fig 1A, 1C and 1H), the TgGT1-infected cells were clearly the most distinct *T*. *gondii* strain along PC2 due to strain differences and/or variation between experiments (TgME49 and TgVEG infections were performed together alone with HhEth1 and HhAmer respectively, while TgGT1 was assayed in a separate experiment. Hierarchical clustering of the distances between samples also further confirmed the differential expression profiles seen in THP-1 cells infected with *H*. *hammondi* as compared to *T*. *gondii* (**Fig 1B**).

We next compared host gene expression of parasite-infected cells to mock-infected cells for all types/strains using *DESeq2* [28] with the thresholds of log_2_ fold-change ≥ 1 or ≤ -1 with *P*_*adjusted (adj)*_ < 0.01 and identified host transcripts that were of higher or lower abundance after infection (S1 Table). A striking difference was observed in the host transcriptional response to *H*. *hammondi* compared to *T*. *gondii*, regardless of strain. Specifically, HhEth1 and HhAmer-infected THP-1 cells had significant changes in ~27% and ~32% of the 15452 queried transcripts, respectively (**Fig 1I** and S1 Table), while in *T*. *gondii* this ranged from ~4% for TgGT1 to ~7% for TgVEG (**Fig 1H** and S1 Table). MA-plots showing average expression vs. log fold-change of each gene further illustrate the stark contrast in the quantitative impact that infection by *T*. *gondii* and *H*. *hammondi* has on the host cell transcriptome (Fig 1C–1E vs. 1F and 1G). We also identified strain-specific gene expression profiles for *T*. *gondii* (as expected based on extensive comparative transcriptomics between members of the type I, II and III lineages) and to a lesser extent, *H*. *hammondi* (Fig 1H and 1I).

### Hyperinduction of inflammatory cytokines by *H*. *hammondi* compared to *T*. *gondii* is recapitulated *in vivo*

**Fig 2:**
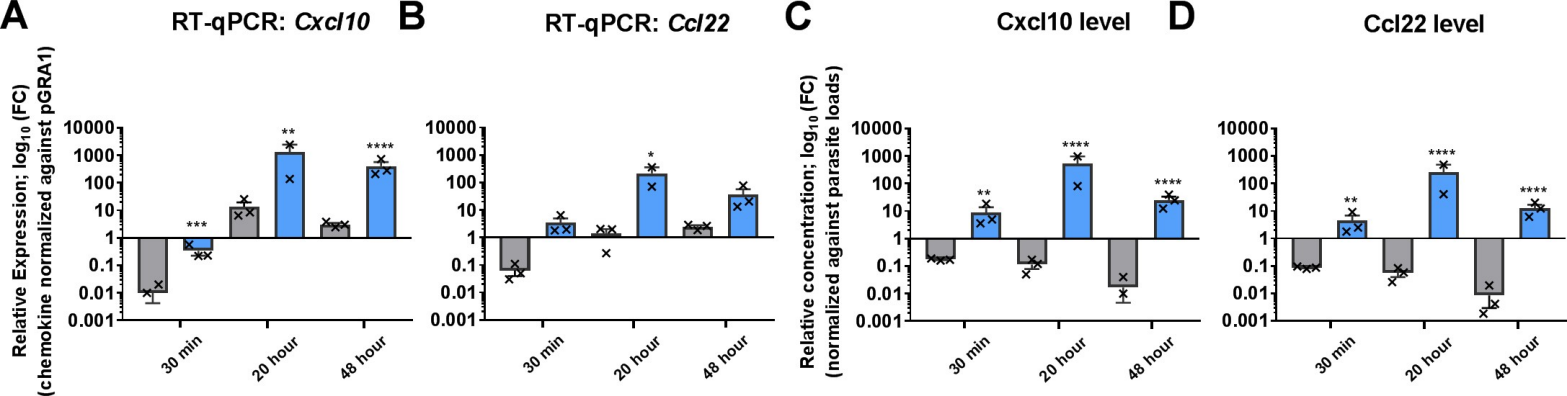
*In vivo H*. *hammondi* infection leads to higher levels of chemokine transcript and protein compared to *T*. *gondii* when normalized to parasite burden. Mice were infected with *T*. *gondii* (TgVEG; grey) or *H*. *hammondi* (HhAmer; blue) or mock-infected with filter-sterilized (0.2 μm) parasite preparations. Mouse peritoneal cell RNA and their supernatants were collected at 30 min, 20 and 48 h post infection. (A-B) Bar graphs show transcript abundance (log_10_) normalized against parasite load (ΔC_T_ for the host gene–ΔC_T_ for *T*. *gondii* or *H*. *hammondi GRA1*). *Cxcl10* transcript abundance normalized in this fashion was significantly higher in HhAmer-infected mice as compared to TgVEG infection at all time points (*p≤0.01) and *Ccl22* transcript abundance was significantly higher in *H*. *hammondi* compared to *T*. *gondii* at 20 h post-infection (*p<0.05). (C-D) Relative concentration of secreted cytokines normalized against parasite loads. Cxcl10 and Ccl22 levels were significantly different in response to TgVEG and HhAmer infections at all time points (**p<0.01, ****p<0.0001). Error bars represent SEM.

While mice are critical intermediate hosts for both *T*. *gondii* and *H*. *hammondi*, little is known about differences in the host response to *T*. *gondii* and *H*. *hammondi in vivo*. Therefore we infected mice intraperitoneally with parasites of each species, collected peritoneal lavage supernatants and peritoneal cells, and quantified cytokine transcript and protein levels. Since *H*. *hammondi* replicates ~4X slower than *T*. *gondii in vitro* [23], we also monitored relative replication rates and parasite loads using species-specific RT-qPCR for the parasite *GRA1* gene (as in [23]; **S1 Fig**). Overall we observed significantly higher levels of host cytokine transcripts and secreted protein in *T*. *gondii*- compared to *H*. *hammondi*-infected mice. However *H*. *hammondi* parasite burden was significantly lower than *T*. *gondii in vivo* (**S1 Fig**) indicating that *H*. *hammondi* replicates more slowly than *T*. *gondii in vivo* consistent with its slower *in vitro* growth phenotype [23]. To compensate for this growth difference we set up an independent experiment where we normalized cytokine transcript and protein level to *GRA1* transcript level, a reasonable proxy for parasite burden [23]. We observed similar cytokine responses in this experiment as the first, and when we normalized cytokine transcript levels (specifically *Cxcl10* and *Ccl22*) in peritoneal cells to *GRA1* transcript level we found that per parasite *H*. *hammondi* infection resulted in significantly higher levels of transcript at 30 min, 20 and 48 h post-infection for *Cxcl10* and 20 h post-infection for *Ccl22* (Fig 2A and 2B). We also observed significantly higher levels of Cxcl10 and Ccl22 protein in peritoneal lavage fluid from *H*. *hammondi*-infected mice compared to *T*. *gondii* (Fig 2C and 2D). These data suggest that the more robust proinflammatory response to *H*. *hammondi* compared to *T*. *gondii* also occurs *in vivo*.

### Pathways altered by *T*. *gondii* and *H*. *hammondi* are mostly shared, with a small number of critical differences

**Fig 3:**
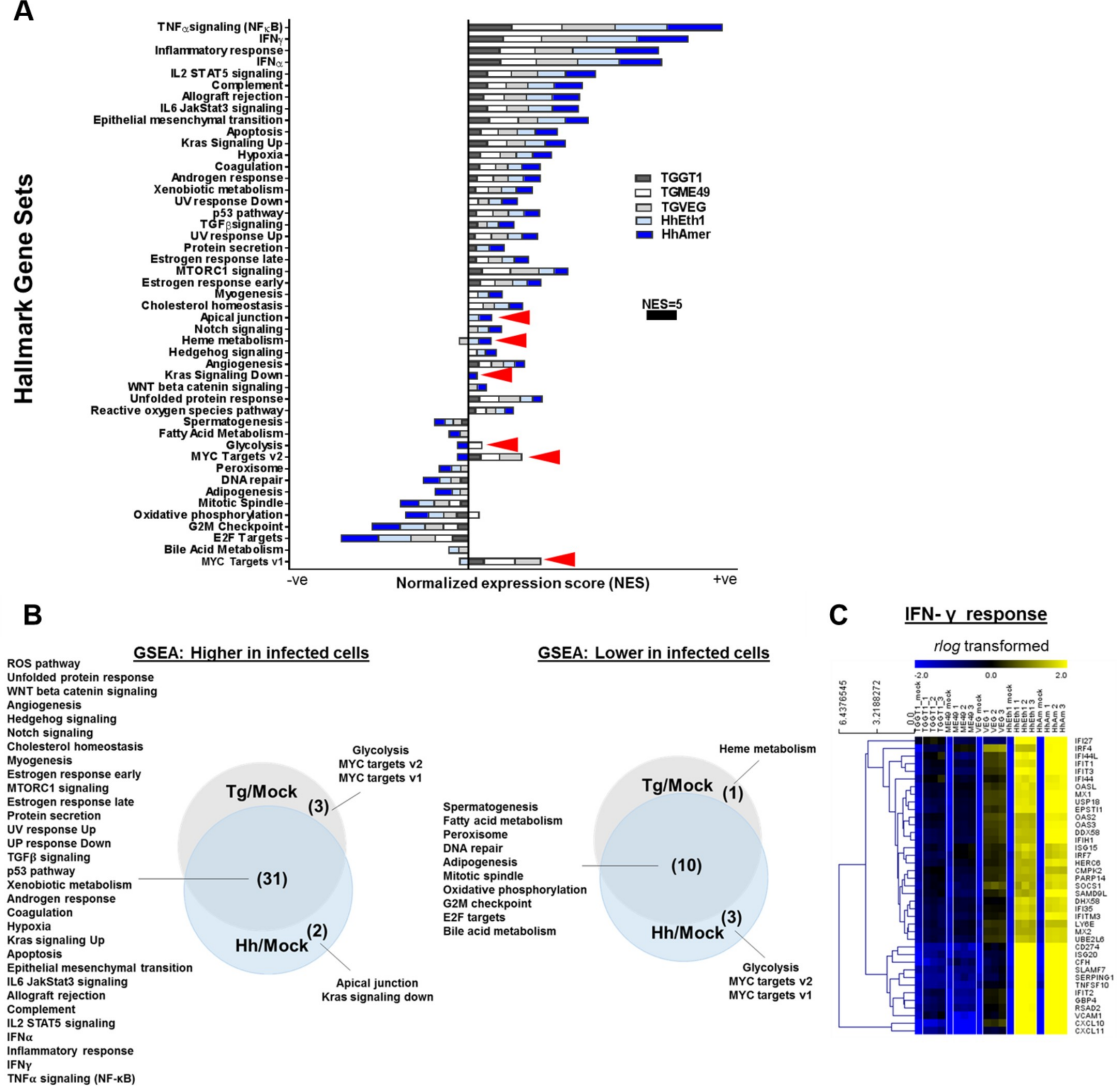
The majority of host pathways altered in *T*. *gondii* and *H*. *hammondi*-infected cells are shared between species. Normalized data was used to perform Gene Set Enrichment Analysis (GSEA) to understand biological relevance of the data set. (A) Bar graph shows normalized enrichment scores (NES) of positively (+ve) and negatively (-ve) enriched curated “Hallmark” gene sets in THP-1 cells in response to *T*. *gondii* (Tg; GT1, ME49 and VEG) and *H*. *hammondi* (Hh; Eth1 and Amer) infections as compared to mock-infected THP-1 cells. Shown are gene sets with a false discovery rate (FDR-q value) <0.05 (computed with 1000 Monte-Carlo simulations). The NES represents the degree to which a gene set is overrepresented at the top or bottom of the ranked list. Bars show stacked NES scores by strain and species to permit comparison. Arrowheads indicate species-specific pathways. (B) Overlapping positive (left panel) and negative (right panel) gene set enrichments summarized in Venn diagrams. (C) Heatmap showing log_2_ expression of a subset of genes from the *IFNγ response* gene set.

While it was clear that *H*. *hammondi*-infected cells displayed a more robust transcriptional change than *T*. *gondii*-infected cells (**Fig 1**), this could occur by differential induction of distinct sets of genes, and/or via differences in the overall magnitude of the induction of the same gene sets. To assess this we used Gene Set Enrichment Analysis (GSEA; [29]) on each transcriptional profile using the curated “Hallmark” gene sets database. Overall we identified 47 gene sets that were significantly enriched in cells infected with *T*. *gondii* and/or *H*. *hammondi* (compared to mock-infection; FDR-q value<0.05), and 41 of these were shared between species (**Fig 3A and 3B**, S1 Table). Interestingly, *H*. *hammondi* infections induced quantitatively higher enrichment of these shared transcriptional changes. For example, for the *IFNγ response* gene set *H*. *hammondi* Eth1 and Amer had normalized enrichment scores (NES) of 11.2 and 11.4 respectively while TgGT1, TgME49 and TgVEG-infected cells had and NES of 8.0, 8.6 and 10.1, respectively (**Fig 3A**, S1 Table). This is perhaps most simply illustrated by a clustered heatmap containing of a subset of the *IFNγ response* gene set, where infection by all strains and species results in increased transcript abundance but the magnitude of that induction is highly species-specific (**Fig 3C**, S2 Table and **S2C Fig**).

We used Ingenuity Pathway Core Analysis (IPA) to identify additional pathways and upstream regulators driving species-specific transcriptional changes. We identified 280 canonical pathways significantly enriched in *T*. *gondii* and/or *H*. *hammondi* infections (*p* <0.01, S3 Table) compared to mock-infected cells including *dendritic cell maturation*, *IL-1 signaling*, *IL-6 signaling* and *interferon signaling* (**S2**A and S2B Fig). Our IPA analysis reaffirm our GSEA results that while *H*. *hammondi* infection alters many of the same canonical pathways and gene sets in THP-1 cells as does infection with *T*. *gondii*, the magnitude of the effect is greater (e.g., *dendritic cell maturation* had *z*-scores of 6.38 and 6.20 for HhAmer and HhEth1 respectively; 5.57, 4.69 and 4.09 for TgGT1, TgME49 and TgVEG respectively; **S2A Fig**, S3 Table). This could be due to 1) *T*. *gondii* suppression of these host responses and/or 2) direct induction of a more dramatic host response during infection by *H*. *hammondi*. Since IFNγ-signaling is critical for controlling *T*. *gondii* infection [30–32] we examined all members of the *interferon signaling pathway* and identified a subset of them (*BAK1*, *1FITM1*, *1FITM2*, *JAK1*, *JAK2* and *PSMB8*) that were of significantly higher abundance in *H*. *hammondi*-infected THP-1 cells but were comparatively unchanged during infection with *T*. *gondii* (**S2B Fig**). Clearly *H*. *hammondi* induces a more robust host response and/or fails to suppress the activation of this and other pathways compared to *T*. *gondii*.

### *T*. *gondii* and *H*. *hammondi*-infected cells have transcriptional profiles indicating distinct cell cycle states

**Fig 4:**
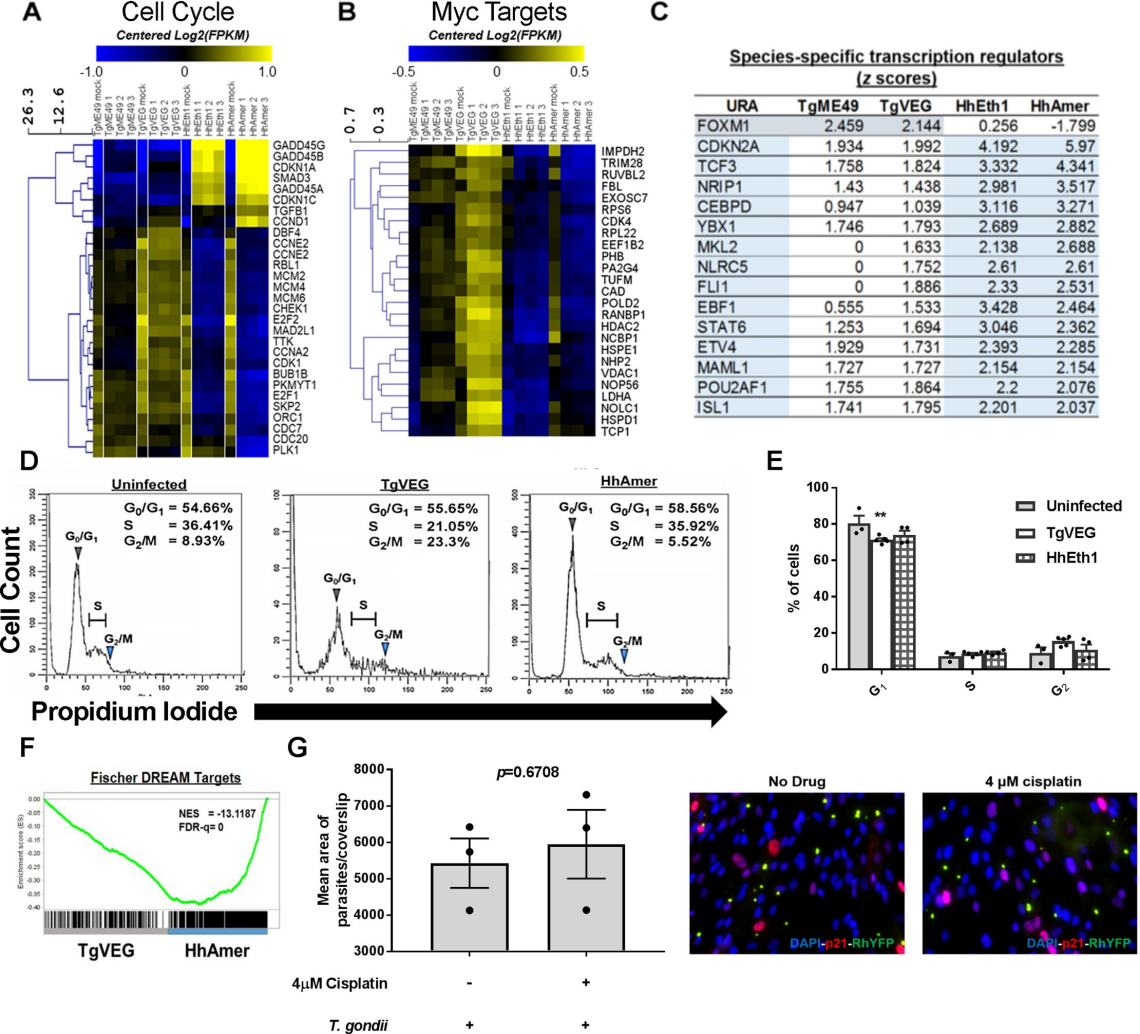
*H*. *hammondi* infection of THP-1 cells leads to a dramatically different impact on cell cycle regulation pathways. (A, B) Heatmaps showing log_2_ transcript abundance for genes belonging to either the Cell cycle (A) or Myc targets v1 (B) gene set across strain and species. Log_2_ normalized data were mean-centered amongst the samples and hierarchically clustered (Euclidean distance). (C) Table showing *z* scores of upstream transcriptional regulators predicted to be activated (*z* scores ≥ 2) and/or inhibited (*z* scores ≤ -2; *T*. *gondii*—grey; *H*. *hammondi*–light blue) in a species-specific manner. (D) THP-1 cells were infected with *T*. *gondii* (TgVEG), *H*. *hammondi* (HhAmer) or mock-treated for 20 hours and fixed with 80% ethanol. THP-1 cells were then stained with propidium iodide (PI) and DNA content was analyzed with flow cytometry. Cell cycle progression was analyzed with ModFitLT software. Black lines represent histograms of DNA content (PI-Area) vs. cell counts. Grey arrows show the G_0_/G_1_ peaks and blue arrows mark the G_2_/M peak. Areas of the histogram in between the G_0_/G_1_ and the G_2_/M peaks contain host cells in the S phase. TgVEG-infected THP-1 cells displayed higher portions of cells found at the G_2_/M phase as compared to HhAmer-infected cells. (E) Cell cycle progression in U2OS cells infected with an MOI of 2 of *T*. *gondii* (TgVEG) or *H*. *hammondi* (HhEth1) was analyzed with propidium iodide (PI) staining and flow cytometry. Percentages of cells in each stage were determined with FlowJo and statistically compared using one way ANOVA followed by Tukey’s post-hoc test. Only *T*. *gondii* VEG-infected, and not *H*. *hammondi* American-infected, cells had a lower percentage of cells in G0/G1 compared to mock-infected cells (**P<0.01). (F) Fisher DREAM Targets gene set enrichment in *H*. *hammondi*-infected cells compared to *T*. *gondii*-infected cells. (G) Growth of *T*. *gondii* Rh-YFP on HFFs in cells treated with cisplatin to induce p21 expression (p21 levels were quantified using immunofluorescence and shown in S4C Fig). HFFs were treated with 4μM of cisplatin or vehicle for 24 h followed by infection with *T*. *gondii* Rh-YFP (MOI = 0.1). *T*. *gondii* Rh-YFP was allowed to grow for 24 h before analyzing growth of the parasites by immunofluorescence (IFA). Numbers of *T*. *gondii* RH-YFP parasites were not significantly different in cisplatin-treated HFFs as compared to mock-treated HFFs (unpaired *t-*test, *p* = 0.67).

Only two gene sets, *Myc targets v1* and *v2*, were significantly enriched in all *T*. *gondii*-infected cells regardless of strain type and either negatively enriched or not significantly enriched *H*. *hammondi*-infected cells (**Fig 3A and 3B**; S3C Fig, bottom panels). Given the fact that *T*. *gondii* is known to induce *c-Myc* translocation into the nucleus and also induces S phase transition in human host cells [33, 34], we wanted to determine if the lack of induction in the *Myc targets v1* and *v2* gene sets in *H*. *hammondi*-infected cells reflected an inability of this species to manipulate cell cycle gene expression and ultimately progression through the cell cycle. We therefore looked specifically at transcript levels for a number of cell cycle and MYC target genes in *T*. *gondii*- and *H*. *hammondi*-infected cells and found multiple E2F-family related genes were of lower abundance in *H*. *hammondi*-infected cells compared to *T*. *gondii*-infected cells (e.g., *E2F1*, *E2F2* and *MCMs 4*, *5*, *6* and *7*; **Fig 4**A and **4**B and S2 Table). This observation is consistent with the lower absolute NES values (for negative enrichment) in *H*. *hammondi* infection compared to *T*. *gondii* strains for the *G2M Checkpoint* and *E2F Targets* gene sets (**Fig 3A**, S1 Table), and suggests that *H*. *hammondi*-infected cells may be in a different cell cycle state. Consistent with this idea, we found that that *H*. *hammondi*-infected cells had significantly higher transcript abundance for *GADD45A*, *B* and *G* (genes involved in DNA damage-induced growth arrest [35, 36]) and *CDKN1A* and *CDKN1C* compared to *T*. *gondii*-infected cells (**Fig 4**A), suggesting that *H*. *hammondi*-infected cells may have a phenotype consistent with DNA damage-induced cell cycle arrest. A role for activation of DNA-damage-mediated pathways is also supported by IPA, in which we identified 38 *H*. *hammondi*-specific pathways including *DNA damage-induced 14-3-3σ signaling* which contains multiple DNA damage-induced cell cycle checkpoint-related genes [37] (**S2B Fig** and S3 Table; highlighted in blue). Combined with the differential *MYC targets* gene sets that were suppressed during *H*. *hammondi* infection compared to *T*. *gondii* (**Figs 3A** and 4B), these data suggest that *T*. *gondii* and *H*. *hammondi*-infections may have a divergent impact on the cell cycle of the host cells that they infect. While *T*. *gondii* induces progression through S phase and into G_2_/M, *H*. *hammondi* infection may lead to cell cycle arrest (in either G_1_/S or G_2_/M) via DNA damage-related stress responses.

### Upstream regulator analysis reveals differential regulation of cell cycle-related transcription factors by *H*. *hammondi* infections

Using IPA upstream regulator analysis, we identified cascades of upstream transcription regulators that might be responsible for driving the observed transcriptional changes after infection with *T*. *gondii* and *H*. *hammondi*. We shortlisted upstream transcription regulators in response to *H*. *hammondi* or *T*. *gondii* infection (compared to mock-infected THP-1 cells) using |*z* scores| ≥ 2 and Fisher’s exact test, *P* <0.01. We identified *FOXM1* as a significant *T*. *gondii*-specific upstream transcription regulator (average Z-score in *T*. *gondii* of 2.31 compared to -0.79 in *H*. *hammondi*), and *CDKN2A* as a *H*. *hammondi*-enriched upstream regulator (average Z-score in *T*. *gondii* of 1.96 compared to 5.08 in *H*. *hammondi*; **Fig 4C**). From the analysis we also identified type/strain-specific and common transcription regulators activated or inhibited in *T*. *gondii* and/or *H*. *hammondi* infections (**Fig 4C**, S4 Table). Common upstream transcription regulators included many well-known signaling mediators involved in *T*. *gondii* infection (e.g. multiple IRFs and STATs including IRF1, 3 and 5 and STAT1) as well as the NFκB p65-encoding gene *RELA* (S4 Table).

### *H*. *hammondi* and *T*. *gondii*-infected THP-1 cells are in distinct phases of the cell cycle

Having shown that *T*. *gondii* and *H*. *hammondi* differentially alter cell-cycle related genes, we analyzed cell cycle progression of sporozoite-infected cells using flow cytometry. Consistent with the literature [33, 34], TgVEG-infected THP-1 cells showed a distinct cell cycle profile compared to uninfected cells, having a significantly higher proportion of cells in G_2_/M (**Fig 4D**). In contrast, the cell cycle profile of HhAmer-infected cells was more similar to uninfected THP-1 cells, with a marked lack of cells in G_2_/M and higher numbers of cells in G_0_/G_1_ phase (**Fig 4D**). Similar results were obtained when comparing THP-1 cells infected with either RH88 tachyzoites or HhAmer sporozoites (**S3A Fig**). We also repeated this experiment in triplicate in U2OS cells and found that cell cycle manipulation differences between *T*. *gondii* and *H*. *hammondi* were not cell-type specific. Specifically, in contrast to *T*. *gondii*-infected U2OS cells which exhibited a prominent G_2_/M peak (**S3B Fig**) and a significantly higher proportion of cells in G2/M compared to uninfected cells (**Fig 4E**), *H*. *hammondi*-infected U2OS cells failed to show this increase in G2/M during infection (**S3B Fig** and Fig 4E).

One mechanism of cell cycle arrest is via DNA damage responses leading to increased CDKN1A transcription leading to increased p21 signaling in the nucleus. *FOXM1* is a component of the FOXM1-MMB complex which is active during G_2_/M and promotes transcription of E2F target genes, while *CDKN2A* (and *CDKN1A* as described above) are both involved in DNA-damage and stress-induced cell cycle arrest via CDK inhibition [35, 38]. Therefore we investigated the possibility that the p53-p21-DREAM pathway was differentially modulated by *T*. *gondii* and *H*. *hammondi* since the DREAM transcriptional repressor regulates a large set of cell cycle-associated genes [39]. We performed GSEA on the *Fischer DREAM Targets* gene set [39] and found that target genes for part of this pathway were suppressed in *H*. *hammondi* infected cells compared to *T*. *gondii* (**Fig 4F**; NES = -13.12, FDR-q = 0). Given the known impact of cell cycle arrest on *T*. *gondii* replication rate [33], and the fact that *H*. *hammondi* replicates slower than *T*. *gondii in vitro* [23] and *in vivo* (**Fig 2**), we postulated that p21-mediated cell cycle arrest might play a role in determining *T*. *gondii* and *H*. *hammondi* replication rate. To test this hypothesis we treated HFFs with 4 μM cisplatin (to induce p21 expression) or vehicle and quantified parasite growth. While cisplatin treated cells had significantly higher nuclear p21 staining compared to vehicle-treated cells (*p* = 0.01; Fig S4C and S4G), *T*. *gondii* infected and proliferated within cisplatin and vehicle-treated cells equally (P>0.05; **Fig 4G**), indicating that p21 upregulation alone was not sufficient to alter *T*. *gondii* replication. These data suggest that the differences in replication rate between *T*. *gondii* and *H*. *hammondi* in HFFs are unlikely due to differences in p21 level.

**Fig 5:**
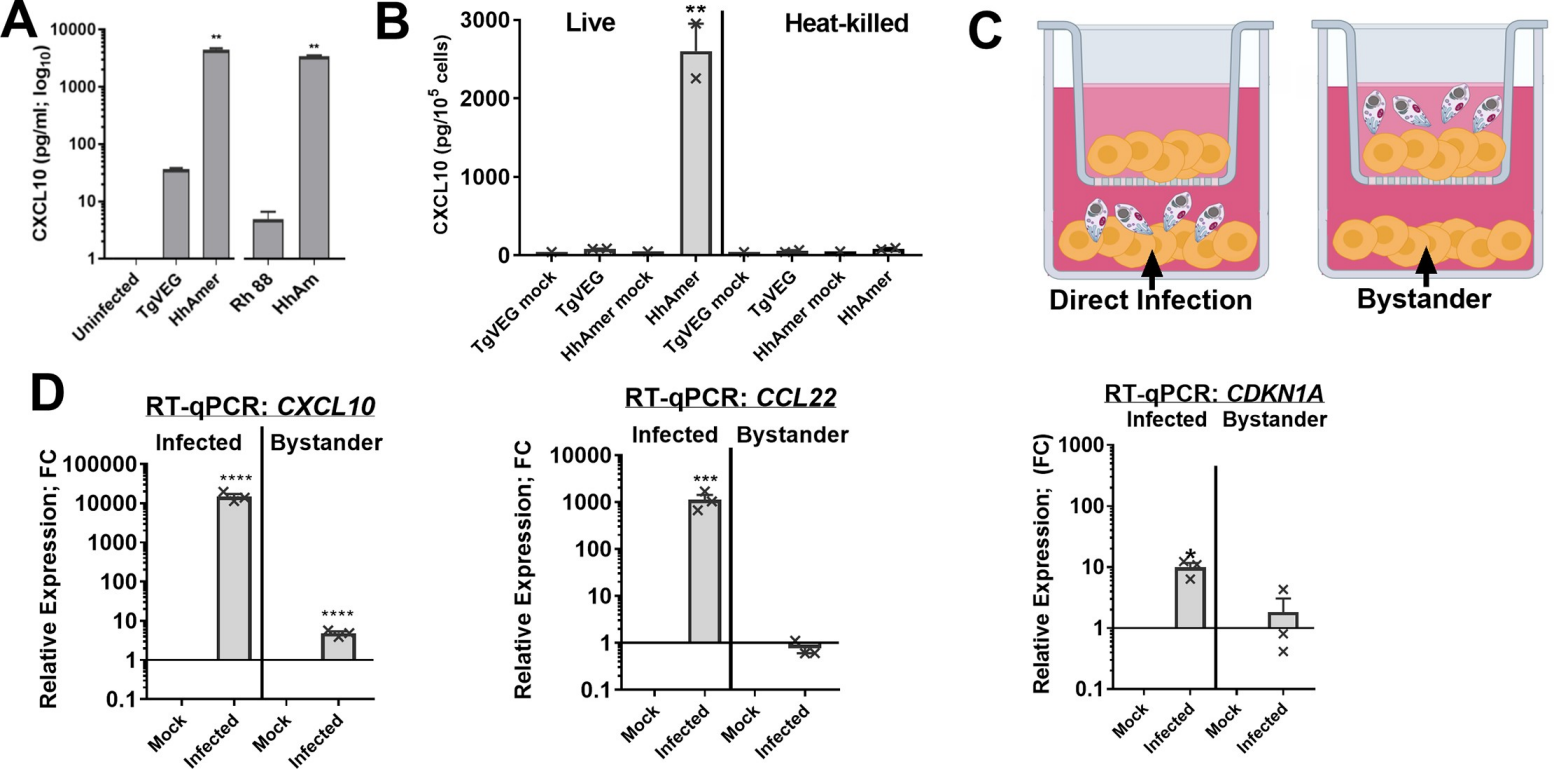
*H*. *hammondi* induction of CXCL10 requires live parasites and can be induced by both direct infection and in bystander cells. (A) CXCL10 secretion in THP-1 cells infected with *T*. *gondii* (Rh88 or TgVEG), *H*. *hammondi* (HhAmer) or mock-treated. THP-1 cells were infected with MOI of 2 of parasites for 20 h and supernatant was collected by pelleting the cells at 200 x g for 10 min. Secretion of CXCL10 was analyzed with ELISA. Secretions of CXCL10 were significantly higher in HhAmer-infected THP-1 cells as compared to mock infection (**p<0.01, ANOVA followed by Tukey’s multiple comparisons test). Data shown in log_10_ scale to indicate some CXCL10 induction above background by *T*. *gondii* infection. (B) TgVEG or HhAmer were heat-killed at 95°C for 5 min and cooled to room temperature before exposing them to THP-1 cells. Heat-killed *H*. *hammondi* fails to induce CXCL10 production by THP-1 cells infected for 24h (actual or equivalent MOI = 2). (C) Schematic for use of transwells in figure panel D, where transcript abundance was quantified in the cells directly infected by parasites (left) or in bystander cells separated by a transwell (right). (D) Direct infection of THP-1 cells with *H*. *hammondi* leads to increased CXCL10 transcript in infected and bystander cells (ANOVA followed by Tukey’s multiple comparisons test, *p<0.01, ***p<0.001 and ****p<0.0001). However, unlike THP-1 cells exposed to direct parasite infection, transcripts of *CCL22* and *CDKN1A* were not significantly higher in bystander cells (*p*>0.05).

Of the chemokines that we quantified at the transcriptional level using RNAseq and the protein level in supernatants of infected THP-1 cells using Luminex Bead Arrays, CXCL10 stood out as having the most *H*. *hammondi*-specific expression phenotype and as shown in **Fig 2** this response could be recapitulated in mice. Therefore we used this chemokine as a sentinel for *H*. *hammondi*-specific responses. To determine if the lack of CXCL10 induction in *T*. *gondii* VEG sporozoite-infected cells was strain- or life-stage-specific, we infected THP-1 cells with TgVEG sporozoites, RH88 tachyozites or with *H*. *hammondi* sporozoites. Consistent with TgVEG, TgRH88 also failed to induce CXCL10 secretion by THP-1 cells (**Fig 5A**), while *H*. *hammondi* infected cells produced ~150-fold more CXCL10 compared to cells infected with either *T*. *gondii* strain (**Fig 5A**). CXCL10 induction required live parasites, in that heat-killed *H*. *hammondi* failed to induce CXCL10 (**Fig 5B**). Since *H*. *hammondi*-infection induced genes related to DNA damage and cell cycle arrest, and since a subset of cell cycle arrest phenotypes (like quiescence regulated by the DREAM complex and cellular senescence) can be transmitted to bystander cells in a paracrine fashion, we used Transwell inserts to separate infected THP-1 cells from bystanders (**Fig 5C**) and quantified *CXCL10*, *CCL22* and *CDKN1A* transcript in both classes of cells. We used CXCL10 and CDKN1A as sentinels for paracrine regulation of senescence and quiescence-associated phenotypes, and CCL22 as a chemokine induced by both parasite species but which is not typically associated with quiescence or senescence associated phenotypes. Since the SASP response can signal other cells in a paracrine manner, we used CXCL10 secretion as a sentinel chemokine for SASP. We found that bystander cells from *H*. *hammondi*-infection had significantly higher *CXCL10* transcript as compared to those from mock infection (by ~10-fold; **Fig 5D**), and RT-qPCR for *H*. *hammondi GRA1* transcript confirmed the absence of parasites in the bystander cells (**S4 Fig**). In contrast to CXCL10, we did not observe any increase in transcript abundance for *CCL22* and *CDKN1A* in bystander cells (**Fig 5D**). CXCL10 was detected in supernatants in both conditions, since this chemokine could freely diffuse after its secretion. Taken together these data provide further support for the hypothesis that *H*. *hammondi* infection, and not *T*. *gondii* infection, leads to a host cell phenotype with hallmarks of quiescent and/or senescent cells capable of altering cell signaling pathways in neighboring cells.

**Fig 6:**
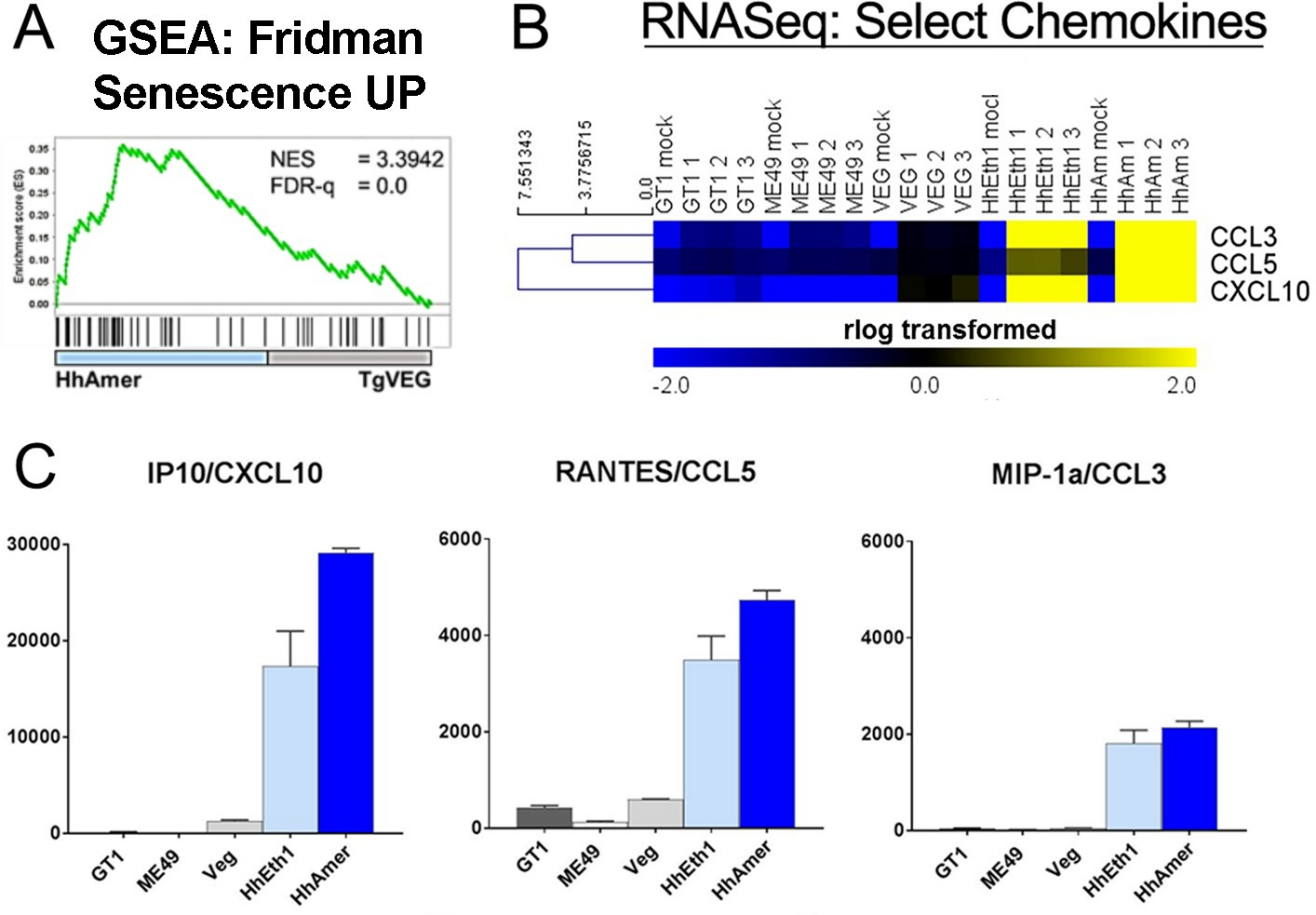
*H*. *hammondi*-infected cells have significantly enriched expression for genes involved in cellular senescence and the senescence-associated secretory phenotype (SASP). (A) GSEA of the *Fridman Senescence Up* gene set in *T*. *gondii* (TgVEG-) and *H*. *hammondi* (HhAmer)-infected THP-1 cells. HhAmer-infected cells were enriched in the *Fridman Senescence Up* gene set with a normalized enrichment score (NES) of 3.4. (B) Heatmap shows mean-centered hierarchically clustered (Euclidean distance) *rlog*-transformed values of transcript abundance for select chemokines and cytokines. (C) Supernatants from THP-1 cells infected with *T*. *gondii* or *H*. *hammondi* were collected 24 h post-infection and analyzed for pro-inflammatory cytokine secretion using the human cytokine 30-plex Luminex kit. Bars show relative changes in fluorescence intensity (fl) in parasite infected cells compared to mock-infected cells (fl infected–fl mock). Proinflammatory cytokines associated with the SASP that were of significantly different abundance between *T*. *gondii* and *H*. *hammondi*-infected THP-1 cells are shown (ANOVA followed by Tukey’s multiple comparisons test, *p*<0.05). Error bars represent SEM of the change in fluorescence units over mock.

Consistent with this observation, the *Fridman Senescence Up* gene set [40] was significantly enriched in *H*. *hammondi*-infected cells with an NES of 3.4 (**Fig 6A**; FDR-q = 0), suggesting that *H*. *hammondi*-infected cells may have some similarities to senescent cells. This would explain the observed differences in the host cell cycle as well as the increased production of inflammatory cytokines via the Senescence-Associated Secretory Phenotype (SASP) [41]. This hypothesis was further supported by IPA analyses showing significant enrichment of genes in the *DNA damage-14-3-3σ signaling* pathway (**S2**A Fig), and the IPA analysis results (**Fig 4**) showing that *CDKN2A* is an important upstream regulator of pathways uniquely altered by *H*. *hammondi* infection and conversely that *FOXM1* signaling targets are enriched in *T*. *gondii*-infected cells (**Fig 4**). One of many defining characteristics of senescent cells is the production of lysosomal senescence-associated β-galactosidase (gal) and β-gal enzyme activity can be detected both in cells and supernatants [42]. We evaluated this in parasite-infected THP-1 cells but found no significant increases in β-gal activity in THP-1 cells infected with either parasite species, even though β-gal activity did increase in Phleomycin-treated THP-1 cells (as a control for β-gal activity; **S5 Fig**). However, we did find that THP-1 cells infected with *H*. *hammondi* had significantly higher levels of IP10/CXCL10, RANTES/CCL5, and MIP-1a/CCL3 transcript compared to *T*. *gondii* (**Fig 6B**), and this increased transcript abundance was also reflected in significantly higher levels of these chemokines in supernatants from *H*. *hammondi*-infected cells (**Fig 6C** and S5 Table). These chemokines are also well-known indicators of the SASP [43].

### *T*. *gondii*-mediated suppression of *H*. *hammondi*-induced CXCL10 is TgIST-dependent

**Fig 7:**
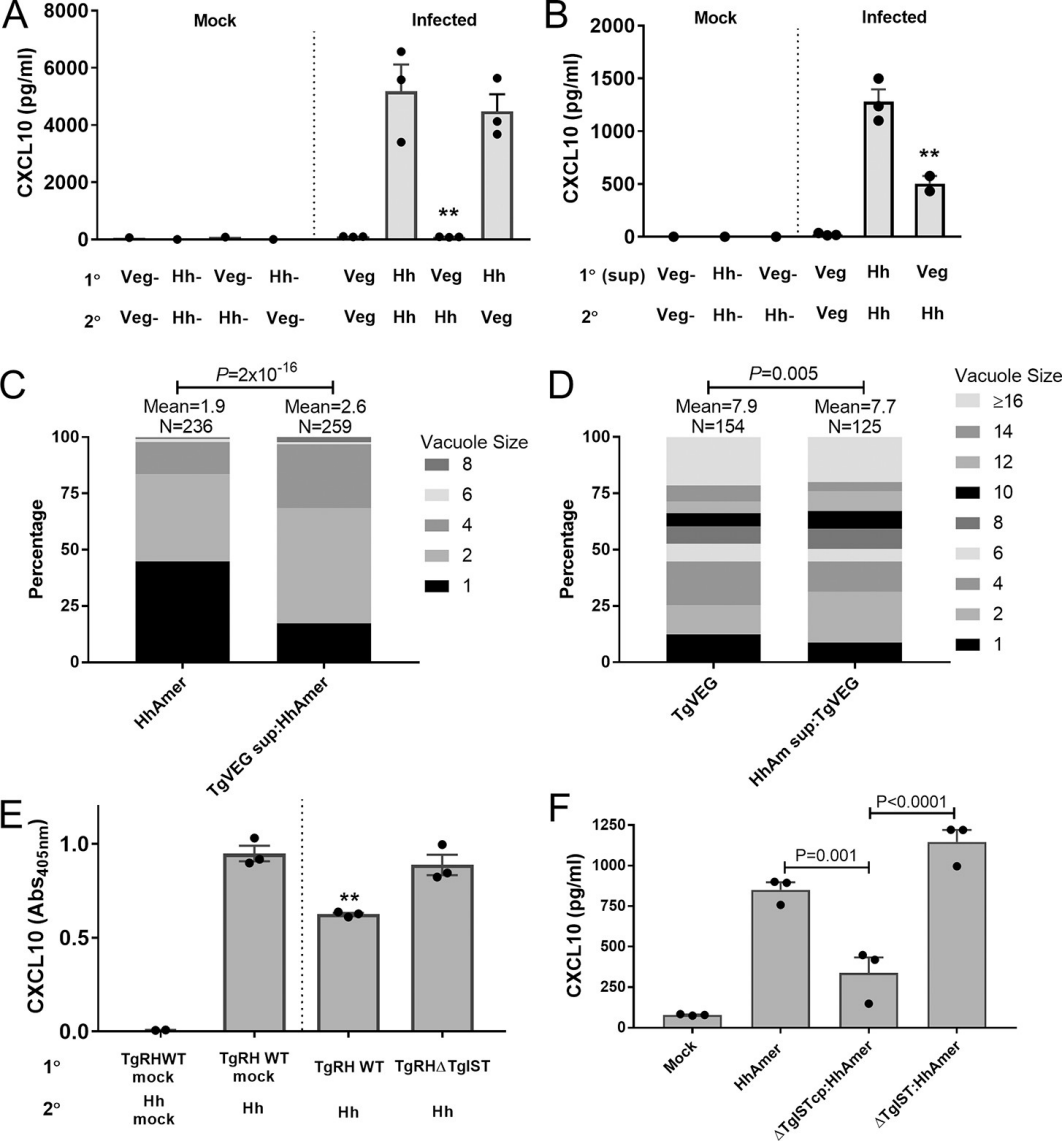
Prior *T*. *gondii* infection suppresses *H*. *hammondi*-mediated host responses and this suppression is partially dependent on *T*. *gondii* IST. (A) THP-1 cells were pre-infected (1°) with freshly excysted *T*. *gondii* (Veg) or *H*. *hammondi* (Hh) sporozoites (MOI = 2). Four hours later, freshly excysted Veg or Hh sporozoites were added into the same wells and CXCL10 levels quantified by ELISA. Prior infection with *T*. *gondii* significantly reduced *H*. *hammondi*-induced CXCL10 production (ANOVA followed by Sidak’s multiple comparison test, **p<0.01) while it was unaffected if the order of infection was reversed (*H*. *hammondi* first, and then *T*. *gondii*). (B) Similar inhibition was observed using *T*. *gondii*-infected THP-1 cell-conditioned medium. Media conditioned with either *T*. *gondii* or *H*. *hammondi*-infected THP-1 cells was prepared by infecting THP-1 cells with freshly excysted Veg or Hh sporozoites (MOI = 2) for 4 h, 0.2 μm filtering the supernatant and then treating fresh THP-1 cells with it for 4 h. Next, these same cells were infected with freshly excysted *T*. *gondii* or *H*. *hammondi* sporozoites (MOI = 2; 2°). Secretion of CXCL10 was significantly lower in THP-1 cells pre-treated with media conditioned with *T*. *gondii*-infected THP-1 cells (**p<0.01, ***p<0.001; ANOVA followed by Tukey’s multiple comparisons test). (C) *T*. *gondii*- or *H*. *hammondi*-infected THP-1 cell conditioned medium generated as in (B) was used to pretreat HFFs for 4 h prior to infection with *H*. *hammondi* (C) or *T*. *gondii* (D) sporozoites. Vacuole sizes were quantified after 24 h of growth. Pre-treatment with *T*. *gondii*-infected THP-1 cell conditioned medium led to a significant (Chi-Squared P-value = 2x10^-16^) increased in *H*. *hammondi* vacuole size, characterized by an increase in 2- and 4-parasite vacuoles compared to mock-treated controls. In contrast, pre-treatment of HFFs with *H*. *hammondi*-infected THP-1 conditioned media caused a smaller, but significant (Chi-Squared P-value = 0.005) decrease in *T*. *gondii* vacuole size. (E) THP-1 cells were infected with wild type *T*. *gondii* strain RH or *T*. *gondii* RHΔIST (MOI = 2) followed by infection 2 h later with freshly excysted *H*. *hammondi* American strain sporozoites (MOI = 2). *H*. *hammondi*-induced secretion of CXCL10 was suppressed by TgWT (**p<0.01; ANOVA followed by Dunnet’s multiple comparisons), but not by RHΔTgIST (*p*>0.05; ANOVA followed by Dunnet’s multiple comparisons). (F) As in (E), prior infection with TgRHΔIST complemented with a functional copy of IST (TgRHΔISTcmp) significantly (P = 0.001) suppressed *H*. *hammondi*-induced CXCL10 secretion, while prior infection with parasites lacking IST (TgRHΔIST) had no significant impact on CXCL10 production.

Next, we speculated that *T*. *gondii* might be capable of suppressing the hyperinflammatory transcriptional and cytokine secretory response by *H*. *hammondi*. To test this hypothesis we exposed THP-1 cells to TgVEG sporozoites for 4 h prior to infection with *H*. *hammondi* (controls were exposed to mock conditions as above) and used ELISA to quantify transcriptional changes across conditions. We found that TgVEG infection significantly and dramatically suppressed *H*. *hammondi*-mediated CXCL10 secretion (**Fig 7A**). However, this suppression only occurred when THP-1 cells were first exposed to *T*. *gondii*, since THP-1 cells pre-infected with *H*. *hammondi* sporozoites followed by *T*. *gondii i*nfection produced similar levels of CXCL10 (**Fig 7A**). In similar experiments, we harvested RNA and subjected it to RNAseq analysis, and found that the suppressive phenotype of prior infection with *T*. *gondii* on *H*. *hammondi* alterations of the abundance of transcripts encoding senescence- and DNA damage-associated gene products (including members of the GADD45 family; **S6 Fig**). Interestingly, suppression of *H*. *hammondi*-induced CXCL10 production also occurred when THP-1 cells were first exposed to *T*. *gondii*/THP-1 conditioned media (harvested 4 h post-infection; **Fig 7B**), suggesting that *T*. *gondii*-mediated suppression is due, at least in part, to parasite- and/or host cell-derived soluble mediators released during infection. However, our data clearly showed that the suppressive effect was most potent when *T*. *gondii* parasites were used as opposed to *T*. *gondii*-conditioned media. Given the dramatic differences in replication rate between *T*. *gondii* and *H*. *hammondi* sporozoites [23], we tested whether *T*. *gondii*/THP-1 conditioned media could alter the division rate of *H*. *hammondi*. Pre-treatment of HFFs with *T*. *gondii*/THP-1 conditioned media resulted in a highly significant (P = 2x10^-16^) increase in *H*. *hammondi* vacuole size (**Fig 7C**). This change was characterized by an increase in the number of 2- and 4-parasite vacuoles (from 38 to 50% and 14 to 28%, respectively; **Fig 7C**). In contrast, when we pre-treated HFFs with *H*. *hammondi*/THP-1 conditioned media, we observed only a small, but significant (P = 0.005; **Fig 7D**) decrease in vacuole size compared to mock-treated controls. Overall these data further show that *T*. *gondii* and *H*. *hammondi* infection induce distinct cellular responses with the capacity to have distinct effects on bystander host cells. Perhaps more importantly they suggest that manipulation of the host cell by *T*. *gondii* may play a role in determining replication rate itself and that this could be exploited to improve cultivation methods for *H*. *hammondi*.

Given the impact of prior *T*. *gondii* infection on CXCL10 secretion we hypothesized that the suppressing factor might be a *T*. *gondii*-secreted protein. Since CXCL10 secretion is induced by IFNγ treatment in a variety of cell types (CXCL10 is also known as interferon-γ-inducible protein 10 or “IP-10” [44]) and *T*. *gondii* IST is known to block IFNγ-induced responses during infection [14, 15], we performed multiple assays with *T*. *gondii* strains lacking TgIST. We found that compared to wild type *T*. *gondii* (RH strain), prior infection with *T*. *gondii* ΔIST parasites was significantly less effective at suppressing *H*. *hammondi*-mediated CXCL10 production (**Fig 7E**). In a separate experiment, we compared the ability of TgΔIST and the same strain complemented with a wild type copy of *T*. *gondii* IST (TgΔISTcmp) to suppress *H*. *hammondi*-induced CXCL10 secretion. Again, we found that THP-1 cells preinfected with TgΔIST parasites secreted significantly more CXCL10 after *H*. *hammondi* infection than those pre-infected with TgΔISTcmp (**Fig 7F**; P<0.0001). While there may be other effectors produced by *T*. *gondii* that are also capable of suppressing CXCL10 and other IFNγ-mediated responses, these data show that the IST effector plays a key role in this phenomenon.

### *H*. *hammondi* fails to suppress IFNγ-induced IRF1 activation and has a significantly less functional IST gene compared to *T*. *gondii*

**Fig 8:**
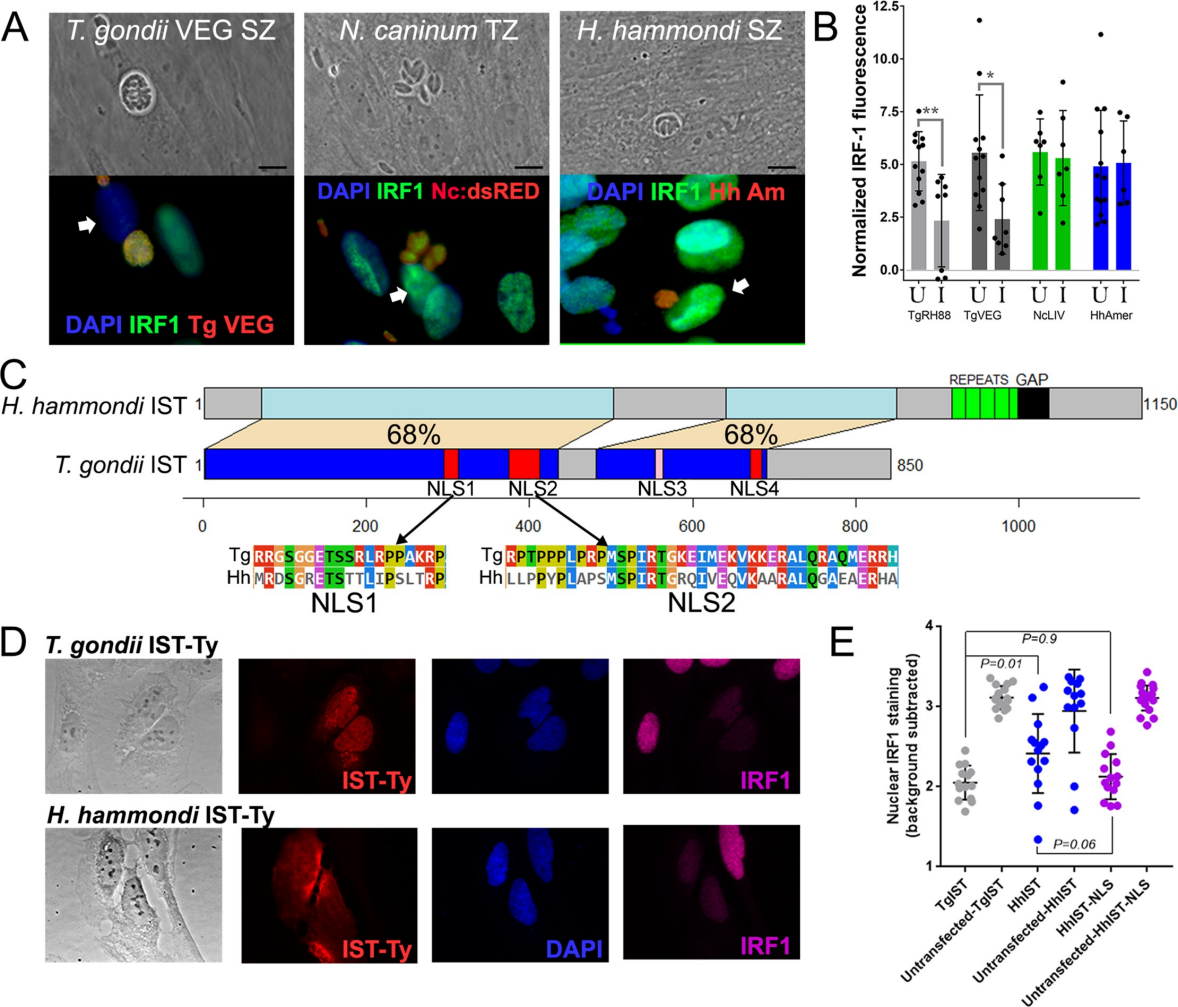
*H*. *hammondi* IST lacks suppressive activity compared to *T*. *gondii* IST due to differences in putative NLS sequences and localization in the host cell. A) In contrast to *T*. *gondii*, *H*. *hammondi* and *N*. *caninum* infection fails to suppress IFNγ-induced IRF1 activation. Representative images are shown for each species, and the white arrowhead indicates the likely nucleus of the infected cell. As expected *T*. *gondii* infected cells have reduced IRF1 staining compared to uninfected neighboring cells while cells infected with *N*. *caninum* or *H*. *hammondi* have similar IRF1 nuclear staining as uninfected bystander cells. (B) Quantification of IRF nuclear staining in cells infected with each indicated species (I) and uninfected bystander cells (U). *T*. *gondii* infection (strains RH88 and TgVEG) significantly reduced IFNγ-induced IRF1 nuclear staining while infection with *N*. *caninum* and *H*. *hammondi* did not. (C) Schematic of the predicted IST protein coding sequence gene from *T*. *gondii* and *H*. *hammondi*, showing conserved (blue and light blue connected by tan) and non-conserved (grey) regions of the predicted proteins. Putative NLS sites are indicated in red or pink, and were only found in *T*. *gondii* IST (not *H*. *hammondi* IST). *H*. *hammondi* has a longer predicted protein that includes repetitive sequence (green) and a sequence assembly gap (black). (D) Ectopic expression of Ty-tagged *T*. *gondii* and *H*. *hammondi* IST in U20S cells showing primarily nuclear staining for TgIST-Ty in contrast to cytoplasmic and nuclear staining for HhIST-Ty. Cell expressing TgIST and HhIST show reduced IRF1 staining in the nucleus after IFNγ treatment. (E) Quantification of nuclear IRF1 staining in IST-expressing cells and neighboring bystanders lacking IST expression. Constructs were TgIST-Ty, HhIST-Ty and HhIST-NLS-Ty (Ty-tagged WT HhIST with a C-terminal NLS). Inclusion of an NLS on the C-terminus of *H*. *hammondi* IST increased its ability to suppress IRF1 induction by IFNγ treatment compared to HhIST-Ty.

Given that *T*. *gondii* IST plays a role in suppressing *H*. *hammondi*-induced CXCL10 production, we hypothesized that this might be due, at least in part, to deficiencies in the function of *H*. *hammondi* IST. TgIST is required for *T*. *gondii* to suppress IFNγ-induced IRF1 nuclear translocation [14, 15], and so we first quantified IRF1 nuclear staining in IFNγ-treated HFFs infected with *H*. *hammondi*, *T*. *gondii*, or *Neospora caninum*. *N*. *caninum* lacks a clear ortholog of TgIST and has been shown previously to be incapable of suppressing IFNγ-induced signaling [15, 45]. As expected, pre-infection of HFFs with TgRH88 tachyzoites or TgVEG sporozoites significantly suppressed IRF1 activation in response to IFNγ treatment (*P*<0.05; **Fig 8A and 8B**), while prior infection with *N*. *caninum* tachyzoites did not (**Fig 8A and 8B**). When the same experiment was performed with *H*. *hammondi* sporozoites, prior infection failed to suppress IFNγ-induced IRF1 activation (**Fig 8A and 8B**), suggesting that sequence differences in the IST locus may determine species-specific differences in the host response. Comparison of the TgIST (TGME49_240060) and HhIST (HHA_240060) predicted protein sequences using BLASTP shows multiple gaps in the alignment and significant differences in the predicted size of the amino acid sequence (**Fig 8C**). While the size difference may be an artifact of sequence assembly breakdown in repetitive sequences found in the C-terminus of HhIST (there is a sequence assembly gap in the C-terminus of HHA_240060; **Fig 8C**; ToxoDB.org), the overall level of identity between TgIST and HhIST in the aligned regions is low (68% for both regions; **Fig 8C**) compared to the predicted proteome-wide average of ~89%. Moreover, *H*. *hammondi* IST lacked any significant predicted nuclear localization sequences, while TgIST harbored multiple predicted NLS’ (3 with a posterior probability >0.5 and one with a posterior probability >0.4; red and pink boxes, respectively; NLStradamus 4 state hidden markov model; [46]). Alignments of the first two predicted NLS sequences between TgIST and HhIST show extensive polymorphism, suggesting that one functional difference between TgIST and HhIST was a lack of NLS targeting sequences. To test this directly, we expressed Ty-tagged forms of TgIST, HhIST and HhIST with a C-terminal NLS in U2OS cells. As expected, based on Ty-tag staining TgIST trafficked primarily to the nucleus of transfected cells and effectively suppressed IFNγ-induced IRF1 activation (**Fig 8D**, top). In contrast, HhIST could be found in the nucleus and cytoplasm of the transfected cell (**Fig 8D**, bottom). When we quantified IRF1 staining in transfected cells and compared it to untransfected cells in the same field of view, we found that HhIST was less effective at blocking IFNγ-induced IRF1 compared to TgIST (P = 0.01; **Fig 8E**). Adding a C-terminal NLS to HhIST partially reversed this deficiency, as HhIST-NLS-expressing host cells had nuclear IRF1 staining intensity that was not significantly different (P = 0.9) from TgIST (**Fig 8E**). These data implicate sequence differences in HhIST in determining deficiencies in the inability of this parasite species to suppress host innate immune responses, and suggest that differences in nuclear localization of IST in the host cell may be at least partially responsible for species-specific differences in the ability to suppress IFNγ-induced signaling.

## Discussion

It is well known that *T*. *gondii* tachyzoites activate a potent host immune response. Despite that, once inside a host cell, *T*. *gondii* is able to modulate, survive and even evade immune responses, all of which lead to replication and ultimate dissemination to distant host tissues. This has largely been attributed to the powerful strategy that *T*. *gondii* employs to co-opt host gene expression [10, 47] and to protect itself from host proteins designed to destroy the parasite-containing vacuole [48, 49]. In contrast little is known about the impact of *H*. *hammondi* on the host cell, and whether its comparatively “avirulent” lifestyle can be attributed at all to differences in the host response. Many of the unique aspects of *H*. *hammondi* biology, including its comparatively slower replication rate, spontaneous (and terminal) cyst formation and inability to be lethal even in IFNγ knockout mice [20], suggest that it is engaged in an inflexible developmental program once sporozoites are released from the oocyst. In the extreme, then, one could hypothesize that the nature and magnitude of the host response may be completely irrelevant to the outcome of infection with *H*. *hammondi*. However our data provide strong evidence to the contrary.

Our study is the first to compare host responses to sporozoites of *T*. *gondii* and *H*. *hammondi*. This particular life stage can be a challenging experimental subject, and batch-to-batch variability in excystation rates and the viability of the obtained sporozoites are typical and have been reported previously [23, 50]. This can likely explain observed differences in the magnitude of host responses to infection as well as differences the *T*. *gondii*-mediated suppressive phenotype (e.g., **Fig 7E** versus **7F**). However, care was taken to use oocysts within 6 months of purification from cat feces and to compare sporozoites to sporozoites whenever possible (e.g., for all experiments involving comparisons between *T*. *gondii* VEG and *H*. *hammondi*).

At first glance, our comparisons of the transcriptomes *T*. *gondii* and *H*. *hammondi*-infected host cells indicate that these parasites modulate the host cell using qualitatively similar strategies. Over 95% of the underlying altered pathways were shared between *T*. *gondii* and *H*. *hammondi*, and fell into categories like inflammation, apoptosis, cell growth, and metabolism that have long been known to be targeted by *T*. *gondii* [1, 47] (**Fig 3**). However when the magnitude of the response was taken into account (whether measured by normalized enrichment scores from GSEA or cytokine secretion), it is clear that *H*. *hammondi*-infected cells are altered more robustly compared to those infected with *T*. *gondii*. This effect, which is also recapitulated in an animal model of infection when differences in parasite burden is taken into account (**Fig 2**), suggests either that a) *H*. *hammondi* has fewer countermeasures to counteract the host response than *T*. *gondii* or b) *H*. *hammondi* has a factor or factors that cause hyperactivation of the immune response (via secreted effectors, and/or production of pathogen- or damage-associated molecular patterns). Regardless of the root causes (which are likely to be complex), the rapid proinflammatory response to *H*. *hammondi* that is not induced and/or suppressed during *T*. *gondii* infection is likely to contribute to dissemination differences between these organisms (e.g., the lack of significant brain tropism in *H*. *hammondi*; [20]).

Intriguingly our results show that the most obvious differential host response in THP-1 cells infected with *T*. *gondii* and *H*. *hammondi* is the regulation of cell cycle (Figs 3, 4 and S4), a phenomenon long-described in *T*. *gondii* but whose role in determining infection outcome is unknown. Unlike *T*. *gondii* infection that actively induces host *c-Myc* [16], the *MYC targets v1* and *v2* gene sets were conversely *suppressed* in THP-1 cells infected with *H*. *hammondi* (Figs 3 and 4). *T*. *gondii* infection leads to an accumulation of cells in the G_2_/M phase [33, 34] and in our data this was accompanied by increased transcript abundance of *FOXM1*-targeted genes (**Fig 4**; [38]). In contrast, *H*. *hammondi* leads to increased transcription of multiple *GADD45* genes which block cell cycle progression as a part of the DNA damage response [35, 38]. DNA damage can induce p53 activation and increase transcription of *CDKN1A* [51], and we observed this outcome **only** in *H*. *hammondi*-infected cells (**Fig 4**A). In addition, our data showed *H*. *hammondi*-mediated suppression of the *p53-DREAM pathway target* gene set which could lead to host cell G1/S cell cycle arrest (**Fig 5A**; [52]). The precise impact of the robust activation of the P53 signaling pathway on *H*. *hammondi* replication *in vitro* is unknown, since experimental manipulation of p21 levels did not alter *T*. *gondii* replication.

Multiple *T*. *gondii* effectors have been implicated in modulating the host cell cycle and levels of host cell cycle regulating effectors like P53 and p21. These include GRA16 [10], which alters P53 levels and parasite virulence, and HCE1/TEEGR [53, 54] which interacts directly with host cyclins and modulates the host cell cycle. The fact that multiple *T*. *gondii* effectors target these overlapping host cell pathways highlights the importance of the replication status of the host cell during *T*. *gondii* infection. The fact that *H*. *hammondi* fails to modulate the host cell cycle in the same fashion as *T*. *gondii* suggests that this may be a derived trait. It is likely that there are functional differences between *T*. *gondii* and *H*. *hammondi* paralogs of GRA16 and HCE1/TEEGR, and now that forward genetics approaches are viable in *H*. *hammondi* [23] this can be tested directly through gene knockouts and/or cross-species complementation experiments.

DNA damage responses can have dramatic effects on cell biology that are independent of cell cycle arrest, and one of these is the Senescence Associated Secretory Phenotype (SASP; [41–43]). We observed increased production of multiple chemokines typically associated with the SASP, including CXCL10 which not only was uniquely present in *H*. *hammondi*-infected supernatants but was also activated in bystander cells via paracrine signaling (**Fig 5**). We did not detect elevated β-gal activity in host cells infected with *H*. *hammondi*, cell types and different cellular conditions could also affect the reliability of any types of senescence markers [41–43]. It is intriguing to postulate that the manipulation of the host cell cycle by *T*. *gondii*, and specifically its ability to induce S-phase transition and ultimately G_2_/M arrest, is a means to prevent the host cell from becoming senescent and activating the SASP in both the infected cell and ultimately in neighboring bystander cells. Disrupting the ability of the host cell to secrete cytokines like CXCL10 would certainly be advantageous given the importance of this chemokine to maintain T-cell populations capable of controlling *T*. *gondii* proliferation [30, 55].

Similar to paracrine effects of the *H*. *hammondi*-induced SASP on bystander cells, *T*. *gondii* induces host- and infection-altering factors to be produced by host cells as well. *H*. *hammondi* has a markedly slow replication rate compared to *T*. *gondii* (shown in multiple studies including [23, 56]), but this slow rate of division appears to be flexible depending upon the host cell environment. The ability to alter the replication of an intracellular parasite like *H*. *hammondi* using *T*. *gondii*-conditioned medium (**Fig 7**) leads to a number of interesting biological questions about the nature and identity of the factors capable of controlling parasite replication. Supernatants from *T*. *gondii*-infected cells have been found to have a variety of effects on recipient cells that vary depending on the donor and recipient cell types (e.g., [57, 58]), although to date the factors themselves have not been identified. Our data provide further compelling evidence for their importance in determining the outcome of *T*. *gondii* infections. In addition to this interesting biology there are additional practical implications of this work with respect to the *H*. *hammondi*/*T*. *gondii* comparative system. The use of *T*. *gondii*-conditioned media may be a first step in further improving culture conditions for *H*. *hammondi*, with the ultimate goal of promoting long term *in vitro* culture which is not yet possible [23, 56].

Finally our data point to a key role for the effector TgIST in suppressing *H*. *hammondi*-induced host responses. While the relevance to infections *in vivo* is currently unknown, it suggests that there is some level of overlap between the pathways that are robustly induced by *H*. *hammondi* and those that are suppressed by *T*. *gondii* IST. Based on our data comparing the ability of *T*. *gondii* and *H*. *hammondi* IST to suppress IFNγ-induced IRF1 activation, these data also suggest that *T*. *gondii* IST has traits that evolved uniquely in this lineage, since they are not shared with *H*. *hammondi* IST and since *N*. *caninum* does not appear to express a functional form of this gene [14, 15]. The lack of predicted nuclear localization signals in *H*. *hammondi* IST as well as its increased functionality in ectopic expression assays suggest that one innovation in TgIST may have been the ability to be robustly trafficked to the host cell nucleus. This may have permitted further optimization of the TgIST gene product to robustly suppress IFNγ induced responses through its interaction with STAT1 targeted genes. This difference in functionality at the TgIST locus may explain some of the well-documented differences in virulence between *T*. *gondii* and *H*. *hammondi*. Regardless, it is possible that the lack of a fully functional *HhIST* gene in *H*. *hammondi* fits with its developmental program. Unlike *T*. *gondii*, *H*. *hammondi* fails to alter its developmental trajectory in the host based on its immune status, and therefore it is possible that it survives the robust immune response that it induces using a pre-programmed period of replication and development. This likely results in lower cyst burdens since it may only replicate and disseminate for 5–7 days, but *H*. *hammondi* cysts are only infectious to cats where even a single cyst can lead to the production of hundreds of millions of oocysts.

### Summary

Comparisons between *T*. *gondii* and its near relatives can shed important new light on the evolutionary origins of pathogenesis in a parasite that has achieved a near global presence in humans and other animals. Here we show that in addition to having dramatic differences in replication rate and development, *T*. *gondii* and *H*. *hammondi* also differ in their abilities to manipulate the host cell. This was somewhat surprising given our prior work showing that pathogenic determinants like ROP18 and ROP5 were functionally conserved in *H*. *hammondi* [22, 24], but we have now identified at least one dense granule protein (IST) that is a molecular determinant of these differences in host cell modulation. Moreover we show for the first time a potential link between species-specific differences in host cell modulation in the intrinsic growth rate of these parasites, and have discovered that *T*. *gondii* may be inducing host cells to produce soluble factors that increase permissiveness to parasite replication in neighboring cells. These discoveries illuminate previously unknown mechanisms of host cell manipulation by *T*. *gondii* that would not have been possible to identify without the use of *H*. *hammondi* as a comparative model. Given that *H*. *hammondi* is a highly successful parasite of animals in its own right [26], the hypothesis emerging from this and our prior work is that *T*. *gondii* and *H*. *hammondi* share nearly all of their genes, but sequence-level divergence at key loci have resulted in the species-specific traits. In the case of *T*. *gondii* these traits led to the emergence of *T*. *gondii* as a highly virulent parasite of animals and humans with a global distribution and the unique ability to dynamically regulate its developmental state based on environmental cues [23].

## Materials and methods

### Cells

Human foreskin fibroblasts (HFFs) and human osteosarcoma bone cells (U2OS) were maintained in cDMEM (100 U/ml penicillin/streptomycin, 100 μg/ml streptomycin, 2 mM L-glutamine, 10% FBS, 3.7 g NaH_2_CO_3_/L, pH7.2; ThermoFisher Scientific). Human monocytes (THP-1) were maintained in cRPMI-1640 (100 U/ml penicillin/streptomycin and 10% FBS; ThermoFisher Scientific) respectively. All cells were grown at 37°C in 5% CO_2_. One day prior to parasite infection, THP-1 cells growth media were replenished with 20% (v/v) of new media (cRPMI). All THP-1 cell infections were performed in cDMEM.

### Mice

Balb/c mice were purchase from Jackson Laboratory and were female aged 6–8 weeks. All animal experiments were approved by the local IACUC at the University of Pittsburgh (Protocol #18032113), with euthanasia conducted according to AVMA guidelines.

### Parasites

Oocysts of *Toxoplasma gondii* (Tg) genotype I (GT1), II (ME49) and III (VEG) and *Hammondia hammondi* (Hh) American (Amer) and Ethiopian-1 (Eth1) isolates were harvested from cat feces 7–11 days after feeding mouse tissues (brain for *T*. *gondii*, leg muscle for *H*. *hammondi*) infected with parasites to 10–20 week old cat free of pathogens [20, 50]. Unsporulated oocysts were isolated via sucrose floatation and placed at 4°C in 2% H_2_SO_4_ to encourage sporulation and for long-term storage. Rh88 tachyzoites were propagated in HFF cells. To obtain pure tachyzoites, HFF cells were washed with PBS, scraped, lysed with 25 gauge needle and filtered with 5 μM syringe-driven filter.

The following knock-out parasite strains were used in this study: RHΔIST [15], RHΔGRA16 [10] and RHΔHCE1 [54]. RHΔHCE1 and RHΔGRA16 are gifts from John Boothroyd. *T*. *gondii* RH88 and RH-YFP tachyzoites and the knock-out parasites were maintained in HFFs cultured in cDMEM. Prior to infection, monolayer with parasites was scraped and syringe lysed with a 25 gauge and a 27 gauge needle, counted with a hemocytometer and added to HFFS, THP-1 or U2OS cells at the desired multiplicity of infection (MOI). Mock-infection was performed by filtering the parasite extracts with a 0.2 μM syringe-driven filter and added the same volume of parasite-free extract to the host cells.

### *T*. *gondii* and *H*. *hammondi* oocyst excystation

We performed oocyst production and excystation as described in detail in [50]. Briefly, sporulated oocysts were washed 3 X in Hank’s balanced salt solution (HBSS; Life Technologies) and treated with 10% bleach (in PBS) for 30 min with shaking at room temperature. Next, bleach was washed off using HBSS and parasites were added to 4 g sterile glass beads (180 μM; Sigma-Aldrich). Oocysts were vortexed on high speed for 15 s on/15 s off for a total duration of 2 min to disrupt the oocyst wall mechanically. Sporocysts were pelleted by centrifugation at 1,000 x g for 10 min. Pellet was resuspended in 5 ml of pre-warmed and freshly made, 0.22 μM filtered excystation media (0.1 g porcine trypsin (Sigma-Aldrich), 2 g Taurocholic Acid (Sigma-Aldrich) in 40 ml PBS, pH 7.5). Sporocysts were incubated in 37°C water bath with 5% CO_2_ for 45 min and syringe-lysed using a 25 and a 22 gauge needle. *H*. *hammondi* sporocysts were syringe-lysed with a 25 gauge needle. To quench the excystation media, 7 ml of cDMEM was added. For some experiments, excysted parasites (sporozoites) were pelleted and resuspended in cDMEM and used to infect monolayers of HFFs overnight at 37°C in 5% CO_2_. HFFs were then scraped, syringe-lysed and filtered with 5 μM syringe-driven filter (Millipore) to obtain pure parasites. In other experiments, freshly excysted sporozoites were used. These included all *in vivo* experiments, experiments analyzing induction of CXCL10 in Transwells, heat-killed parasite assays and parasite replication assays. Within an experiment, all *T*. *gondii* VEG and *H*. *hammondi* sporozoites were treated the same, and in all cases parasites were used within 24 hr of excystation.

### Human monocyte cell line infection

Prior to infection, THP-1 cells were seeded at 1 x 10^5^ cells/well in 24-well plates in cDMEM. To prepare parasites for the infections, HFFs monolayers containing parasite sporozoites were scraped, syringe-lysed, and pelleted (in some cases, freshly excysted oocysts were used). After resuspending the pellet in cDMEM, the parasite mixture was filtered through a 5 μM syringe-driven filter (Millipore). THP-1 cells were infected with either *T*. *gondii* or *H*. *hammondi* at multiplicity of infection (MOI) of 4, 2 or 1.6. Three biological replicates were made for each parasite infection. THP-1 cells were also mock-infected with parasite filtered through a 0.22 μM syringe-driven filter (Millipore). These protocols are described in detail in [50].

### RNA isolation

RNA was collected at 24 h post-infection or 20 h post-secondary infection (for coinfection experiments) from parasite- and mock-infected cells using the RNeasy Kit according to the manufacturer instructions (Qiagen). QIAShredder spin columns (Qiagen) were used to homogenize the samples prior to RNA extraction and contaminating DNA was degraded using RNase-free DNase (Qiagen). RNA was eluted in 50 μl of RNase-free water and gel electrophoresis and Nanadrop RNA quantification were performed to ensure RNA integrity. One mock and three biological infection replicates were done for all experiments. Total RNA samples were kept at -80°C.

### mRNA-sequencing and data processing

mRNA-sequencing libraries and Illumina next generation sequencing were performed at the Core Facility at the University of Pittsburgh. Integrity of the RNA was analyzed with Agilent 2100 Bioananalyzer and all purified RNA samples had RIN scores >9. RNA was sequenced using NextSeq 550 (Illumina) and were pooled and sequenced over four lanes. Strand-specific, 150 bp, single-end RNA-sequencing was performed. Read libraries were mapped to the human genome (*Homo sapiens* ensemble v81; hg38) and transcriptome (*Homo sapiens* ensemble v81; hg38) with default options on CLC Genomics Workbench v11.0. Fastq files have been deposited in the NCBI short read archive (Accession number: SRX3734421-8).

### Differential expression analysis using *DESeq2* and Pre-ranked Gene Set Enrichment Analysis

*DESeq2* [28] was used to perform differential expression analysis of genes in THP-1 cells infected with *T*. *gondii* and *H*. *hammondi*. Raw reads (total gene reads; exported from CLC Genomics Workbench) with at least 1 read count in all samples were analyzed. Prior to analyzing differential gene expression, integrity of the data was examined using principal component analysis (PCA) and distances of all samples were calculated (embedded in the *DESeq2* package). Genes were considered to be differentially expressed in THP-1 cells if the log_2_ fold-change was ≥ 1 or ≤ -1 and with a *P*_*adj*_ value (alpha) <0.01.

Pre-ranked Gene Set Enrichment Analysis (GSEA; [29]) was performed to compare gene sets that were enriched in THP-1 cells in relation to parasite infections. Ranked list calculating the fold-change difference (subtracting normalized log_2_ fold-change of infected host cells from mock infected host cells) was used for the analysis.

### Ingenuity Core Pathway Analysis

Core analysis from Ingenuity Pathway Analysis (IPA; Qiagen) was used to examine biological relevance of the RNA-seq data. Canonical pathways and upstream regulators that are over-represented in *T*. *gondii* and *H*. *hammondi* infection were analyzed using log_2_ fold-change and *p*_adj_ value obtained in *DESeq2* [28]. Only genes that were deemed significantly expressed were used in the analysis (log_2_ fold-change ≥ 1 or ≤ -1 and *p*_adj_ value < 0.01 for Infected vs. Mock). IPA default settings were used for the analysis. For both canonical pathway and upstream regulator analysis, pathways and genes with activation *z* scores ≥ 2 were deemed to be activated while activation *z* scores ≤ -2 were deemed to be inhibited for different analysis. Threshold for *p* value significant used in the analysis was <0.05 or–log(p-value) >1.3.

### Infected mouse peritoneal cell collection

Mouse infections were performed in two independent experiments. In the first, 40,000 sporozoites (TgVEG or HhAmer) were grown overnight in HFFs prior to being harvested by needle passage and filtering, and resuspended in PBS. Parasites were intraperitoneally injected into 7–9 week old mice. Mice were euthanized with CO2 according to IUCAC protocols and dissected. Peritoneal cells were collected in 3 ml PBS as described previously [23]. The second experiment was identical except that the dose was 20,000 per mouse, and instead of culturing parasites overnight after excystation they were used immediately.

### Multiplex cytokine analysis

THP-1 cells were infected with MOI 4 of *T*. *gondii* or *H*. *hammondi* (same infection set up as the RNA-seq experiments). THP-1 cells infected or mock-infected with parasites were pelleted at 1,000 x g for 10 min. Supernatant were collected and stored at -80°C. Luminex was performed at the Luminex Core Facility University of Pittsburgh Cancer Institute. Each sample was measured (fluorescence intensity) in duplicate.

### Cytokine analysis using ELISA

Concentrations of the human pro-inflammatory cytokine CXCL10 and CCL22 and mouse Ifnγ, Il12p40, Cxcl10 and Ccl22 in mouse peritoneal cell supernatants were analyzed by ELISA according to the manufacturers instructions (Human and Mouse DuoSet ELISA respectively, R&D Systems). For relative chemokine concentration in comparison to parasite load, log_2_ values of the absolute concentration of chemokines was calculated and relative transcript abundance in relation to parasite burden was calculated using the 2^-ΔΔCT^ method (ΔΔC_T_ = log_2_ chemokine concentration–ΔC_T_ *GRA1*).

### Transwell and heat-killed parasite infections

Freshly excysted TgVEG or HhAmer was added to THP-1 cells seeded in 24-well plates (control) or added onto Transwell inserts (0.4 μM polycarbonate membrane, Corning). For heat-killed parasite infection, freshly excysted sporozoites were heat-killed at 95°C for 5 min and cooled to room temperature before infecting THP-1 cells. Multiplicity of infection of 2 was used in these experiments. Supernatants were collected either by pelleting the cells or collected from the bottom chamber of the Transwell setup.

### Reverse transcriptase-quantitative PCR (RT-qPCR)

RNA were extracted using RNeasy RNA extraction kit as above and according to manufacturer’s instructions (Qiagen). cDNA was reverse transcribed from 1 μg of RNA using SuperScript IV First-Strand synthesis system (ThermoFisher Scientific). RT-qPCR was performed using QuantStudio with SYBR Green (Applied Biosystems). The PCR mixture contained 1 X SYBR Green buffer (BioRad), 0.25 μl of forward and reverse primers (S6 Table) and cDNA. Genes were amplified using the standard protocol (95°C for 10 min and 40 cycles of 95°C for 15 sec and 60°C for 1 min). Data were acquired and analyzed using QuantStudio^TM^ Design & Analysis Software (ThermoFisher Scientific) and data exported to Microsoft Excel for threshold values (C_T_) and 2^-ΔΔCT^ for fold change analysis. GAPDH and *Gapdh* was used as the human and mouse reference gene respectively (control) for the 2^-ΔΔCT^ analysis. For *in vivo* 2^-ΔΔCT^ analysis, relative expression of target genes were normalized against the ΔC_T_ of mock-infected mice. For relative expression normalized by comparison with parasite load, we used *GRA1* as a reference gene for parasite burden. To do this, the ΔC_T_ of target genes and *GRA1* were first obtained (C_T_ target gene or *GRA1 –*C_T_ *Gapdh*). Then, ΔΔC_T_ target gene was calculated (ΔC_T_ target genes–ΔC_T_ *GRA1)*. Relative expression was finally calculated using the 2^-ΔΔCT^ methods for respective target genes. Primers validation and melt curve analysis were performed to ensure integrity of the RT-qPCR. No RT and water controls were also included in every RT-qPCR.

### Immunofluorescence

All IFAs were performed in HFFs seeded on glass coverslips. Infected cover slips were fixed with 4% paraformaldehyde in PBS for 20 min. Cover slips were rinsed once with 1X PBS and permeabilized with IFA buffer (PBS and 0.2% Triton-X). Cover slips were then blocked with the blocking buffer (PBS, 0.2% Triton-X and 5% BSA) for 30 min. All these steps were performed at room temperature (RT). *CDKN1A/p21* was detected using the mouse monoclonal antibody p21 (Santa Cruz Biotechnology) either overnight at 4°C or for 1 h at RT. Secondary anti-mouse antibody conjugated to Alexa Fluor 488, or 647. Cover slips were mounted with DAPI (4’,6-diamidino-2-phenylindole) stain (Vector Laboratories). Fluorescence was detected using fluorescence microscope (Olympus) and images were analyzed using ImageJ or CellSense. The same exposure time was used for any given comparison data set. Growth of RH-YFP was determined by analyzing area of parasite growth from at least five field of views (FOV) from a single coverslip. Results were obtained from three separate coverslips.

### Cell cycle analysis

Cell cycle profiles of THP-1 cells were analyzed with propidium iodide staining according to manufacturers instructions (ThermoFisher Scientific). THP-1 cells were infected with *T*. *gondii* or *H*. *hammondi* at an MOI of 2. For uninfected cells, THP-1 cells were seeded at the same time as the other cells in different treatment groups and an equal volume of media was added to the uninfected cells in place of parasite infection. At 20 h post-infection, cells were pelleted and washed 1X with chilled PBS and fixed in chilled 80% ethanol at -20°C for overnight. Cells were then washed with chilled PBS and rehydrated for 15 min with chilled PBS. Cells were stained with 2 μg/mL propidium iodide/RNaseA (ThermoFisher Scientific) solution for 15 min at room temperature with gentle agitation. Stained cells were analyzed immediately by flow cytometry using LSR II (BD Biosciences) at the United Flow Core at University of Pittsburgh or kept at -20°C in the dark for no longer than 24 hr until flow cytometry analysis. Cell cycle profiles were analyzed with ModFit LT 5.0 (VSH).

### β-galactosidase activity assay

β-galactosidase activity was analyzed in THP-1 cells and secreted proteins using the β-galactosidase assay kit (ThermoFisher) according to manufacturers instructions. Briefly, THP-1 cells were inoculated with *T*. *gondii* or *H*. *hammondi*, treated with 1 μg/mL of phleomycin in cDMEM. At 72 h post-infection, cells were pelleted at 300 x g for 10 min and supernatant was collected for β-galactosidase activity assay. The cell pellets were washed 2 times with PBS and lyzed with IP lysis buffer (ThermoFisher) with Halt Protease Inhibitor Cocktail (ThermoFisher), before analyzing for β-galactosidase activity. β-galactosidase activity was determined after 1 h of incubation at 37°C and absorbance was read at 420nm.

### IFNγ-induced IRF1 expression derived from parasite infection

HFFs grown on coverslips were infected with TgVEG and *H*. *hammondi* sporozoites (MOI 0.5) for 72 hours and with RH88 and *N*. *caninum* (NC-1) (MOI 0.5) for 3 hours prior to treatment with IFNγ. Infected cells were treated with recombinant human IFNγ protein (1 ng per mL of cDMEM; Gibco PHC4031) for 15 hours. Infected host cells were fixed with 4% paraformaldehyde. Coverslips were stored in blocking buffer (5% BSA, 0.1% Triton X-100) at 4°C until staining was performed. Coverslips were stained with goat *Toxoplasma gondii* Polyclonal Antibody (1:500) (Invitrogen PA1-7256) and rabbit IRF1 (1:200) (Cell Signaling Technology, 8478S) primary antibodies for 1 hour at room temperature. Coverslips were washed 3X with PBS and stained with Goat-anti Rabbit IgG Alexa Fluor 488 (1:1000) (Invitrogen A-11008) and Donkey anti-Goat IgG Alexa Fluor 594 (1:1000) (Invitrogen A-11058) secondary antibodies for 1 hour at room temperature. Coverslips were washed 3X with PBS and mounted with Prolong Diamond antifade mountant with DAPI (Invitrogen P36962).

### IFNγ-induced IRF1 expression derived from mammalian expression

U2OS cells were grown on coverslips in cDMEM supplemented with 10% FBS and 2mM L-glutamine. For transfections, cells were maintained in cDMEM supplemented with only 2mM L-glutamine. Cells were transfected with 500ng of plasmid DNA using the Lipofectamine 3000 Transfection Reagent (Invitrogen L3000001) according to the manufacturer’s instructions. Cells were incubated at 37°C, 5% CO_2_ for 17 hours. Cells were treated with IFNγ recombinant human protein (1 ng per mL of cDMEM; Gibco PHC4031) for 6 hours. Coverslips were fixed with 4% paraformaldehyde and stored in PBS at 4°C until staining was performed. Coverslips were incubated with blocking buffer (5% BSA, 0.15% Triton X-100) for 1 hour and stained with mouse Ty1 Tag monoclonal Ab (BB2) (1:100) (Invitrogen MA5-23513) and rabbit IRF1 (1:200) (Cell Signaling Technology, 8478S) primary antibodies for 1 hour at room temperature. Coverslips were washed 3X with PBS and stained with Goat-anti Rabbit IgG Alexa Fluor 647 (1:1000) (Invitrogen A-21244) and Goat anti-Mouse IGG Alexa Fluor 594 (1:1000) (Invitrogen A-11032) secondary antibodies for 1 hour at room temperature. Coverslips were washed 3X with PBS and mounted with Prolong Diamond antifade mountant with DAPI (Invitrogen P36962).

### Ethics statement

Work described herein involves animal research, all of which was approved by the local IACUC committee at both the USDA and University of Pittsburgh under protocol numbers 15–017 (USDA) and 18032113 (U. Pittsburgh). All animals were euthanized according to AVMA guidelines. For felines, this involved the use of a sedative followed by lethal barbiturate injection, and for mice this involved exposure to CO_2_ in a closed chamber at a flow rate between 30%-70% of the chamber volume according to AVMA guidelines.

## Supporting information

S1 Fig *T. gondii* VEG (TgVEG) and *H. hammondi* Amer (HhAmer) sporozoite infections in vivo. Mice were infected intraperitoneally with *T*. *gondii* (TgVEG; grey) or *H*. *hammondi* (HhAmer; blue). Mice were also mock-infected with filter-sterilized (0.2 μm) parasite preparations. Mouse peritoneal cell RNA was collected at 30 min, 20 and 48 h post-infection and mRNA levels of parasite *GRA1* were quantified using RT-qPCR as a proxy for parasite abundance, using mouse *Gapdh* as the reference gene. (A) *T*. *gondii* or *H*. *hammondi GRA1* transcript abundance relative to mouse *Gapdh* transcript abundance in mice infected with 20,000 freshly excysted TgVEG or HhAmer sporozoites. Bar graphs show ΔC_T_ (C_T_ *GRA1* –C_T_ *Gapdh*) at three different time points post-infection. Parasite burdens of HhAmer are lower than TgVEG (smaller ΔC_T_ values observed in mice infected with TgVEG; Sidak’s multiple comparisons test **p<0.01 and ****p<0.0001 within each time point). (B) Table showing C_T_ values of mice infected with *T*. *gondii* or *H*. *hammondi* at indicated time points.
(PDF)

S2 Fig Top canonical pathways enriched (*z* scores) in *T. gondii* or *H. hammondi*-infected THP-1 cells compared to mock-infected cells. Log_2_ fold-change and DESeq2-derived *p*_*adj*_ values of genes with differential transcript abundance (log_2_ fold-change ≥ 2 or ≤ -2; *p*_*adj*_ < 0.01) were analyzed by Ingenuity Pathways Analysis®. **(Top Panel)** Stacked bars show pathways significantly enriched in infected cells compared to mock-treatment (-log_10_(p-value) > 1.3 = *p* < 0.05; *z* scores ≥ 2 or ≤ -2). Positive *z* scores represent pathways that were enriched in infected cells and negative *z* scores represent pathways enriched in uninfected cells. **(Bottom panel)** The majority of pathways enriched in infected or mock-treated cells were shared between *T*. *gondii* and *H*. *hammondi* infection. Exceptions for each species are indicated. ***T*. *gondii* and *H*. *hammondi*-infected cells share many similar activated pathways but with significant quantitative differences. (Left panel)** THP-1 cells infected with *T*. *gondii* or *H*. *hammondi* have significantly higher levels of the majority of transcripts belonging to the Canonical Interferon signaling pathway (from IPA), suggesting that this pathway is targeted by and/or responds to both species. **(Right Panel)** Heatmap of transcript abundance (log_2_-transformed) from TgVEG and HhAmer-infected THP-1 cells for some members of the Interferon signaling pathway illustrates species-specific transcript abundances. Infected cells shown are represented by the solid boxes while mock-infected cells represented by striped boxes. Data were mean-centered and hierarchically-clustered (Euclidean distance). **Enrichment plots from Gene Set Enrichment Analysis (GSEA) of THP-1 cells infected with *T*. *gondii* or *H*. *hammondi*.** Enrichment plots showing GSEA results for *IFNγ response* (top panels) and *MYC targets v1* gene sets (bottom panels) in THP-1 cells after infection with *T*. *gondii* (Tg) or *H*. *hammondi* (Hh). Rank is based on log2-transformed normalized transcript abundance in infected cells compared to mock. Normalized enrichment scores (NES) are also indicated for each of the plots. The *IFNγ response* gene set was highly positively enriched in response to *T*. *gondii* and *H*. *hammondi* infection while MYC targets v1 gene set was positively enriched in *T*. *gondii*-infected cells and negatively enriched in *H*. *hammondi*-infected cells.
(PDF)

S3 Fig (A,B) Representative histograms showing cell cycle progression in THP-1 (A) and U2OS (B) cells infected with *T*. *gondii* (Rh88 or TgVEG) or *H*. *hammondi* (HhAmer or HhEth1) for 20 hours. Both host cell types showed increased percentages of cells in G2/M after infection with *T*. *gondii*, while *H*. *hammondi* infection led to more cells in G0/G1 and fewer in G2/M compared to uninfected cells. (C) Quantification of p21 immunofluorescence in 4μM cisplatin-treated HFFs compared to mock-treated HFFs. Cisplatin treatment significantly increased nuclear p21 staining intensity in HFFs compared to vehicle alone (*p = 0.0136).
(PDF)

S4 Fig Bystander cells lack any detectable expression of parasite transcript. C_T_ values of *H*. *hammondi* GRA1 transcript detection in infected and bystander THP-1 cells cells (*H*. *hammondi* added to cells in the Transwell insert) N.D., Not Detected.
(PDF)

S5 Fig β-galactosidase (gal) assay to detect activity of β-gal in THP-1 cells (Pellet) and supernatant. THP-1 cells were infected with *T*. *gondii* (TgVEG) or *H*. *hammondi* (HhAmer) sporozoites with an MOI of 1.6 for 72 h and cells and supernatants were collected to quantify β-gal activity. THP-1 cells were also treated with 50 μg/uL phleomycin for 72 h to induce senescence and β-gal secretion as a positive control. β-gal activity was significantly higher in the phleomycin-treated THP-1 cells as compared to untreated THP-1 cells, while neither TgVEG nor HhAmer infection significantly altered secreted or cell-associated β-gal activity (Tukey’s multiple comparisons test, *p*>0.05).
(PDF)

S6 Fig Heatmap of log2(FPKM) RNAseq data from THP-1 cells infected with *H. hammondi*, *T. gondii* strain VEG, or pre-infection with one species followed by infection with the other (HV→H. hammondi and then T. gondii; VH→T. gondii VEG followed by H. hammondi). Genes shown were mean-centered and then hierarchically clustered. Genes shown are a subset of the “Fridman Senescence UP” gene set as described in the manuscript and arrowheads indicate key genes including cyclin-dependent kinases and DNA damage response genes such as members of the GADD45 family. These data suggest that prior infection with *T*. *gondii* suppresses the ability of *H*. *hammondi* to induce DNA damage response pathways in the host cell.
(PDF)

S1 Table DESeq2 output for comparisons between THP-1 cells infected with different parasite strains and species and mock-treated THP-cells. (XLSX)

S2 Table GSEA datasets found to be statistically significant for each strain and/or species comparison between infected and mock-treated THP-1 cells. (XLSX)

S3 Table Log2 (FPKM) transcript count values across all THP-1 samples infected with different strains and species (or mock-infected) for genes belonging to either the *IFNg* Response or *MYC Targets V1* gene sets from the Hallmarks GSEA database. (XLSX)

S4 Table Ingenuity Pathway Analysis output for THP-1 cells infected with *T. gondii* or *H. hammondi* or subjected to mock treatment. (XLSX)

S5 Table Signficant “upstream regulators” of downstream targets modulated by infection with T. gondii or H. hammondi compared to mock-treated THP-1 cells. (XLSX)

S6 Table Backround-subtracted fluorescence intensity values of 30 target analytes found in supernatants from infected or mock-treated THP-1 cells using Luminex bead arrays. (XLSX)

S7 Table Sequences of primers used in this study. (DOCX)
