# Supplementary material for: Head-to-head comparisons of Toxoplasma gondii and its near relative Hammondia hammondi reveal dramatic differences in the host response and effectors with species-specific functions
Source: PLoS Pathog. 2020 Jun 23;16(6):e1008528. doi: 10.1371/journal.ppat.1008528 (PMC7360062; doi:10.1371/journal.ppat.1008528)
Supplement: S1 Fig — Mice were infected intraperitoneally with T. gondii (TgVEG; grey) or H. hammondi (HhAmer; blue). Mice were also mock-infected with filter-sterilized (0.2 μm) parasite preparations. Mouse peritoneal cell RNA was collected at 30 min, 20 and 48 h post-infection and mRNA levels of parasite GRA1 were quantified using RT-qPCR as a proxy for parasite abundance, using mouse Gapdh as the reference gene. (A) T. gondii or H. hammondi GRA1 transcript abundance relative to mouse Gapdh transcript abundance in mice infected with 20,000 freshly excysted TgVEG or HhAmer sporozoites. Bar graphs show ΔCT (CT GRA1 –CT Gapdh) at three different time points post-infection. Parasite burdens of HhAmer are lower than TgVEG (smaller ΔCT values observed in mice infected with TgVEG; Sidak’s multiple comparisons test **p<0.01 and ****p<0.0001 within each time point). (B) Table showing CT values of mice infected with T. gondii or H. hammondi at indicated time points. (PDF) [file ppat.1008528.s001.pdf]

SUPPLEMENTARY FIGURES

A

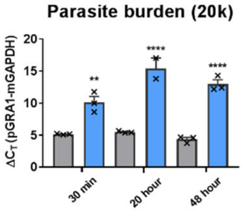


B

| | Time | 30 min | | | | 20h | | | | 48h | | | |
|---|---|---|---|---|---|---|---|---|---|---|---|---|---|
| | | Mock | Infected | | | Mock | Infected | | | Mock | Infected | | |
| *Gapdh* | TgVEG | 18.790 | 17.678 | 17.829 | 17.382 | 17.303 | 16.493 | 18.267 | 16.346 | 17.555 | 14.817 | 15.354 | 15.292 |
| | HhAmer | 18.141 | 17.040 | 18.214 | 17.593 | 17.459 | 17.388 | 17.677 | 21.673 | 16.030 | 14.930 | 17.188 | 15.752 |
| *Gra1* | TgVEG | u.d. | 22.867 | 22.905 | 22.627 | u.d. | 22.185 | 23.684 | 21.728 | u.d. | 18.595 | 19.966 | 19.979 |
| | HhAmer | u.d. | 28.811 | 26.845 | 27.584 | u.d. | 31.192 | 34.723 | u.d. | u.d. | 29.246 | 29.382 | 28.162 |

u.d. = undetermined

**S1 Fig. *T. gondii* VEG (TgVEG) and *H. hammondi* Amer (HhAmer) sporozoite infections *in vivo*.** Mice were infected intraperitoneally with *T. gondii* (TgVEG; grey) or *H. hammondi* (HhAmer; blue). Mice were also mock-infected with filter-sterilized (0.2 µm) parasite preparations. Mouse peritoneal cell RNA was collected at 30 min, 20 and 48 h post-infection and mRNA levels of parasite *GRA1* were quantified using RT-qPCR as a proxy for parasite abundance, using mouse *Gapdh* as the reference gene. (A) *T. gondii* or *H. hammondi GRA1* transcript abundance relative to mouse *Gapdh* transcript abundance in mice infected with 20,000 freshly excysted TgVEG or HhAmer sporozoites. Bar graphs show $\Delta C_T$ ($C_T$ *GRA1* – $C_T$ *Gapdh*) at three different time points post-infection. Parasite burdens of HhAmer are lower than TgVEG (smaller $\Delta C_T$ values observed in mice infected with TgVEG; Sidak's multiple comparisons test $**p<0.01$ and $****p<0.0001$ within each time point). (B) Table showing $C_T$ values of mice infected with *T. gondii* or *H. hammondi* at indicated time points.
