# Supplementary material for: Head-to-head comparisons of Toxoplasma gondii and its near relative Hammondia hammondi reveal dramatic differences in the host response and effectors with species-specific functions
Source: PLoS Pathog. 2020 Jun 23;16(6):e1008528. doi: 10.1371/journal.ppat.1008528 (PMC7360062; doi:10.1371/journal.ppat.1008528)
Supplement: S2 Fig — Log2 fold-change and DESeq2-derived padj values of genes with differential transcript abundance (log2 fold-change ≥ 2 or ≤ -2; padj < 0.01) were analyzed by Ingenuity Pathways Analysis®. (Top Panel) Stacked bars show pathways significantly enriched in infected cells compared to mock-treatment (-log10(p-value) > 1.3 = p < 0.05; z scores ≥ 2 or ≤ -2). Positive z scores represent pathways that were enriched in infected cells and negative z scores represent pathways enriched in uninfected cells. (Bottom panel) The majority of pathways enriched in infected or mock-treated cells were shared between T. gondii and H. hammondi infection. Exceptions for each species are indicated. T. gondii and H. hammondi-infected cells share many similar activated pathways but with significant quantitative differences. (Left panel) THP-1 cells infected with T. gondii or H. hammondi have significantly higher levels of the majority of transcripts belonging to the Canonical Interferon signaling pathway (from IPA), suggesting that this pathway is targeted by and/or responds to both species. (Right Panel) Heatmap of transcript abundance (log2-transformed) from TgVEG and HhAmer-infected THP-1 cells for some members of the Interferon signaling pathway illustrates species-specific transcript abundances. Infected cells shown are represented by the solid boxes while mock-infected cells represented by striped boxes. Data were mean-centered and hierarchically-clustered (Euclidean distance). Enrichment plots from Gene Set Enrichment Analysis (GSEA) of THP-1 cells infected with T. gondii or H. hammondi. Enrichment plots showing GSEA results for IFNγ response (top panels) and MYC targets v1 gene sets (bottom panels) in THP-1 cells after infection with T. gondii (Tg) or H. hammondi (Hh). Rank is based on log2-transformed normalized transcript abundance in infected cells compared to mock. Normalized enrichment scores (NES) are also indicated for each of the plots. The IFNγ response gene set was highly posit [file ppat.1008528.s002.pdf]

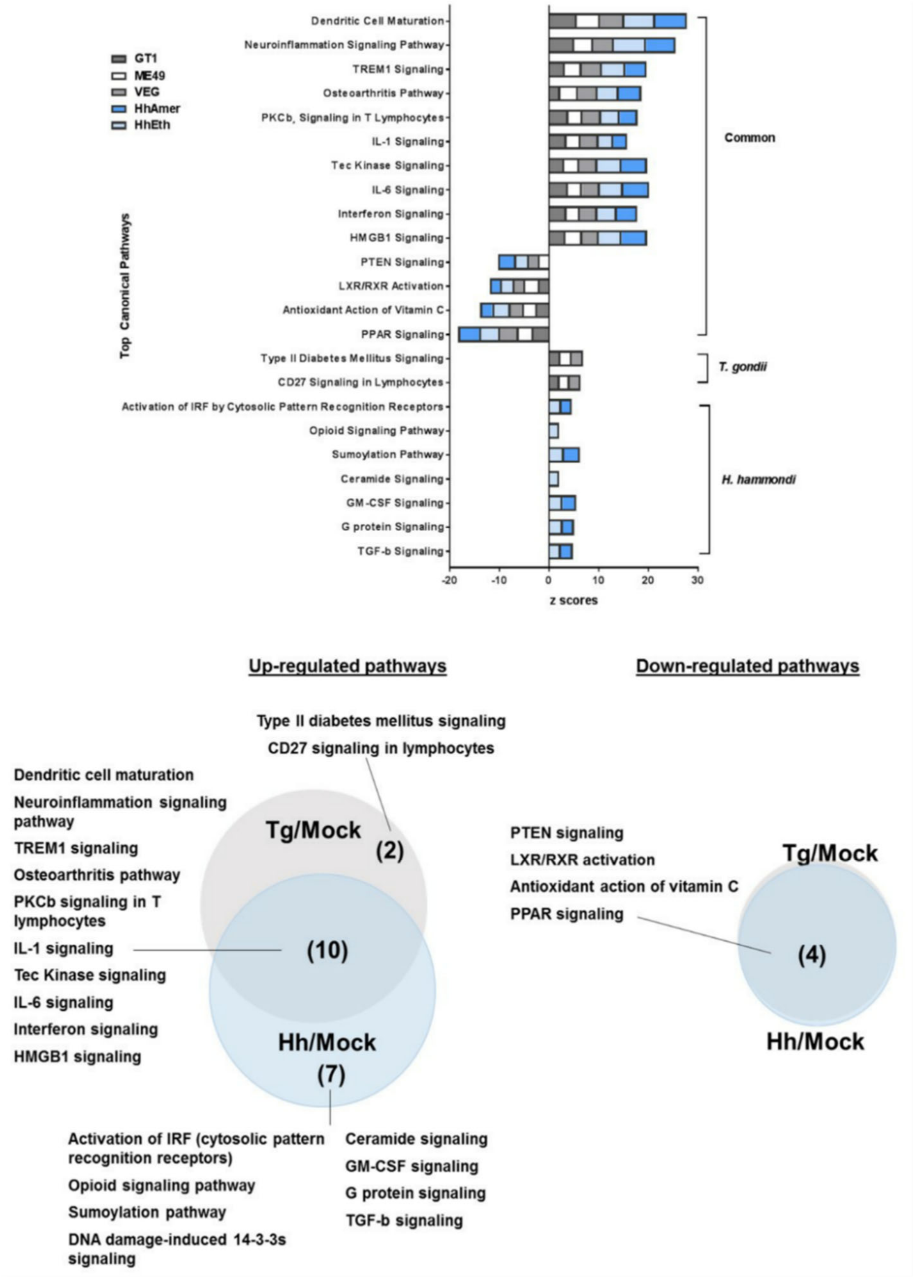

S2A Fig. Top canonical pathways enriched (*z* scores) in *T. gondii* or *H. hammondi*-infected THP-1 cells compared to mock-infected cells. Log_2 fold-change and DESeq2-derived p_{adj} values of genes with differential transcript abundance (log_2 fold-change ≥ 2 or ≤ -2; $p_{adj} < 0.01$) were analyzed by Ingenuity Pathways Analysis®. **(Top Panel)** Stacked bars show pathways significantly enriched in infected cells compared to mock-treatment ($-log_{10}$(p-value) > 1.3 = $p < 0.05$; z scores ≥ 2 or ≤ -2). Positive z scores represent pathways that were enriched in infected cells and negative z scores represent pathways enriched in uninfected cells. **(Bottom panel)** The majority of pathways enriched in infected or mock-treated cells were shared between *T. gondii* and *H. hammondi* infection. Exceptions for each species are indicated.

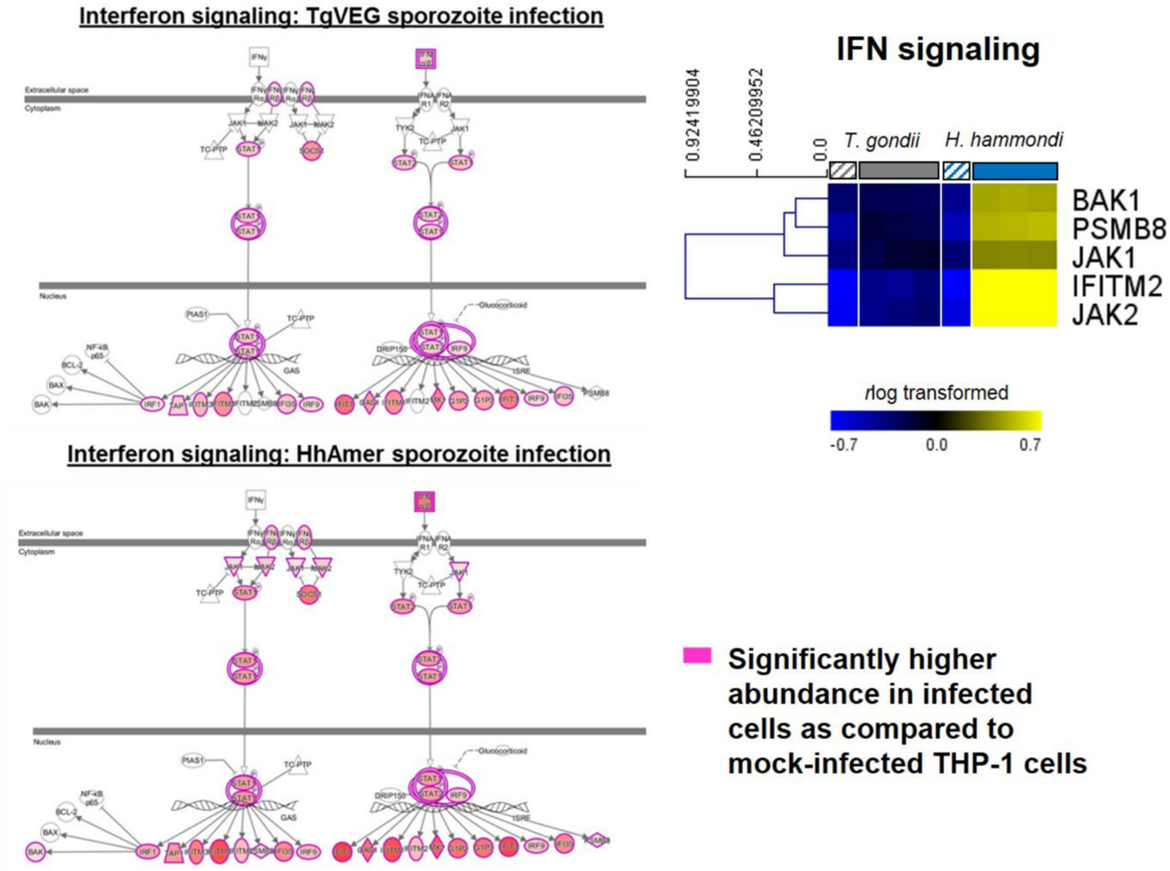

S2B Fig. *T. gondii* and *H. hammondi*-infected cells share many similar activated pathways but with significant quantitative differences. (Left panel) THP-1 cells infected with *T. gondii* or *H. hammondi* have significantly higher levels of the majority of transcripts belonging to the Canonical Interferon signaling pathway (from IPA), suggesting that this pathway is targeted by and/or responds to both species. **(Right Panel)** Heatmap of transcript abundance (log_2-transformed) from TgVEG and HhAmer-infected THP-1 cells for some members of the Interferon signaling pathway illustrates species-specific transcript abundances. Infected cells shown are represented by the solid boxes while mock-infected cells represented by striped boxes. Data were mean-centered and hierarchically-clustered (Euclidean distance).

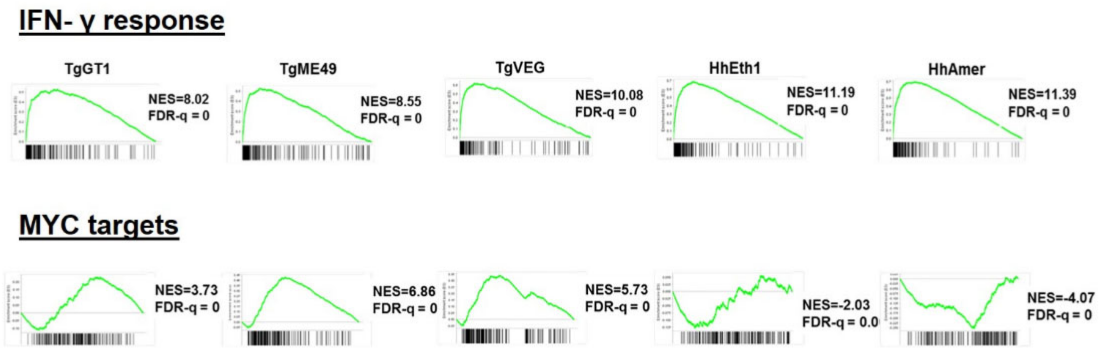

S2C Fig. Enrichment plots from Gene Set Enrichment Analysis (GSEA) of THP-1 cells infected with *T. gondii* or *H. hammondi*. Enrichment plots showing GSEA results for *IFNγ response* (top panels) and *MYC targets v1* gene sets (bottom panels) in THP-1 cells after infection with *T. gondii* (Tg) or *H. hammondi* (Hh). Rank is based on log2-transformed normalized transcript abundance in infected cells compared to mock. Normalized enrichment scores (NES) are also indicated for each of the plots. The *IFNγ response* gene set was highly positively enriched in response to *T. gondii* and *H. hammondi* infection while MYC targets v1 gene set was positively enriched in *T. gondii*-infected cells and negatively enriched in *H. hammondi*-infected cells.
