# Supplementary material for: Head-to-head comparisons of Toxoplasma gondii and its near relative Hammondia hammondi reveal dramatic differences in the host response and effectors with species-specific functions
Source: PLoS Pathog. 2020 Jun 23;16(6):e1008528. doi: 10.1371/journal.ppat.1008528 (PMC7360062; doi:10.1371/journal.ppat.1008528)
Supplement: S3 Fig — (A,B) Representative histograms showing cell cycle progression in THP-1 (A) and U2OS (B) cells infected with T. gondii (Rh88 or TgVEG) or H. hammondi (HhAmer or HhEth1) for 20 hours. Both host cell types showed increased percentages of cells in G2/M after infection with T. gondii, while H. hammondi infection led to more cells in G0/G1 and fewer in G2/M compared to uninfected cells. (C) Quantification of p21 immunofluorescence in 4μM cisplatin-treated HFFs compared to mock-treated HFFs. Cisplatin treatment significantly increased nuclear p21 staining intensity in HFFs compared to vehicle alone (*p = 0.0136). (PDF) [file ppat.1008528.s003.pdf]

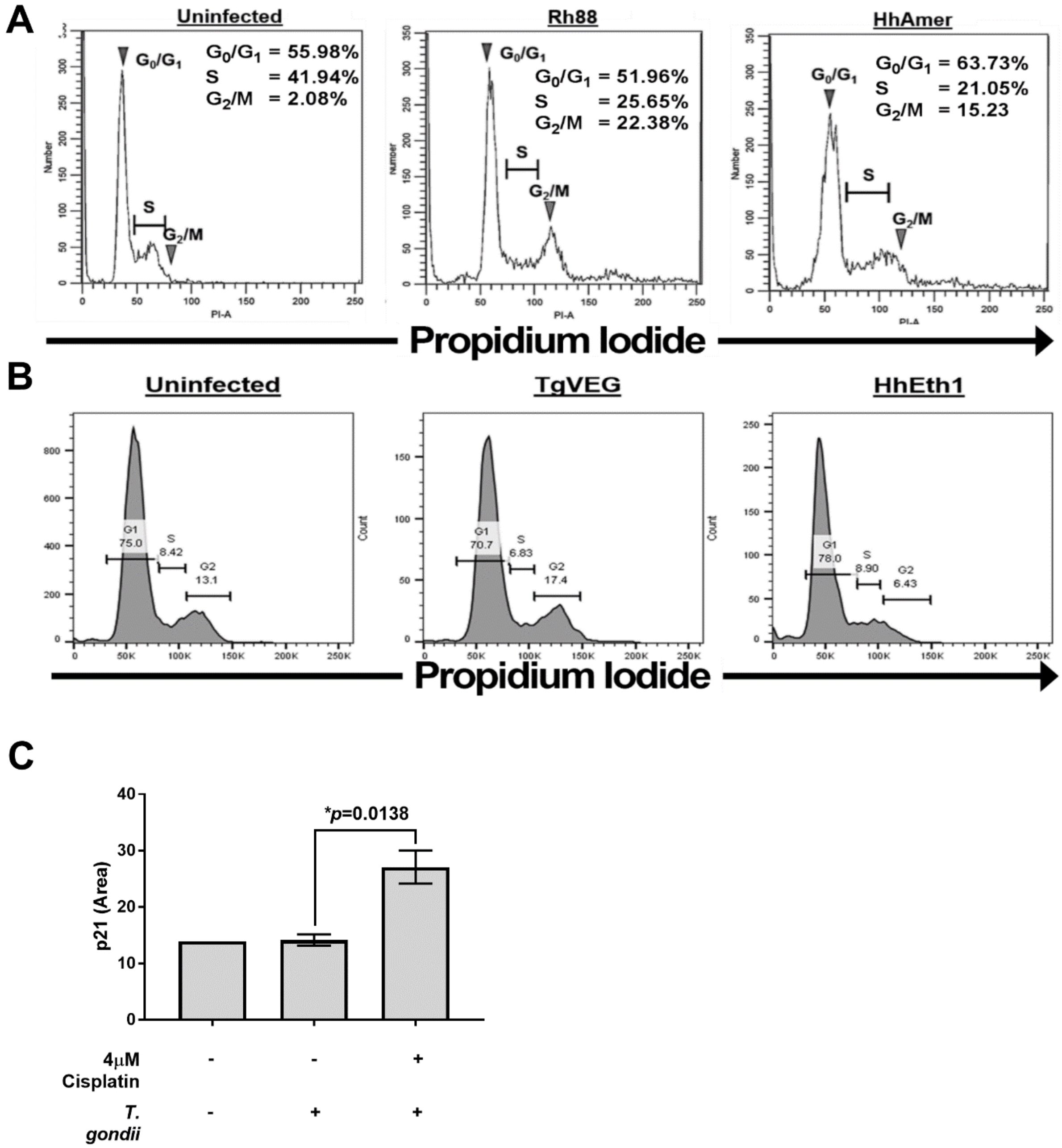

S3 Fig. (A,B) Representative histograms showing cell cycle progression in THP-1 (A) and U2OS (B) cells infected with *T. gondii* (Rh88 or TgVEG) or *H. hammondi* (HhAmer or HhEth1) for 20 hours. Both host cell types showed increased percentages of cells in G2/M after infection with *T. gondii*, while *H. hammondi* infection led to more cells in G0/G1 and fewer in G2/M compared to uninfected cells. (C) Quantification of p21 immunofluorescence in 4μM cisplatin-treated HFFs compared to mock-treated HFFs. Cisplatin treatment significantly increased nuclear p21 staining intensity in HFFs compared to vehicle alone (*p=0.0136).
