# Supplementary material for: Head-to-head comparisons of Toxoplasma gondii and its near relative Hammondia hammondi reveal dramatic differences in the host response and effectors with species-specific functions
Source: PLoS Pathog. 2020 Jun 23;16(6):e1008528. doi: 10.1371/journal.ppat.1008528 (PMC7360062; doi:10.1371/journal.ppat.1008528)
Supplement: S4 Fig — CT values of H. hammondi GRA1 transcript detection in infected and bystander THP-1 cells cells (H. hammondi added to cells in the Transwell insert) N.D., Not Detected. (PDF) [file ppat.1008528.s004.pdf]

Experiment	Sample	*H. hammondi GRA1*
Transwell®	Mock	N.D
	Infected_1	N.D
	Infected_2	N.D
	Infected_3	N.D
Control	Mock	N.D
	Infected_1	22.39
	Infected_2	22.58
	Infected_3	22.74

S4 Fig. Bystander cells lack any detectable expression of parasite transcript. C_T values of *H. hammondi* GRA1 transcript detection in infected and bystander THP-1 cells cells (*H. hammondi* added to cells in the Transwell insert) N.D., Not Detected.
