# Supplementary material for: Head-to-head comparisons of Toxoplasma gondii and its near relative Hammondia hammondi reveal dramatic differences in the host response and effectors with species-specific functions
Source: PLoS Pathog. 2020 Jun 23;16(6):e1008528. doi: 10.1371/journal.ppat.1008528 (PMC7360062; doi:10.1371/journal.ppat.1008528)
Supplement: S5 Fig — THP-1 cells were infected with T. gondii (TgVEG) or H. hammondi (HhAmer) sporozoites with an MOI of 1.6 for 72 h and cells and supernatants were collected to quantify β-gal activity. THP-1 cells were also treated with 50 μg/uL phleomycin for 72 h to induce senescence and β-gal secretion as a positive control. β-gal activity was significantly higher in the phleomycin-treated THP-1 cells as compared to untreated THP-1 cells, while neither TgVEG nor HhAmer infection significantly altered secreted or cell-associated β-gal activity (Tukey’s multiple comparisons test, p>0.05). (PDF) [file ppat.1008528.s005.pdf]

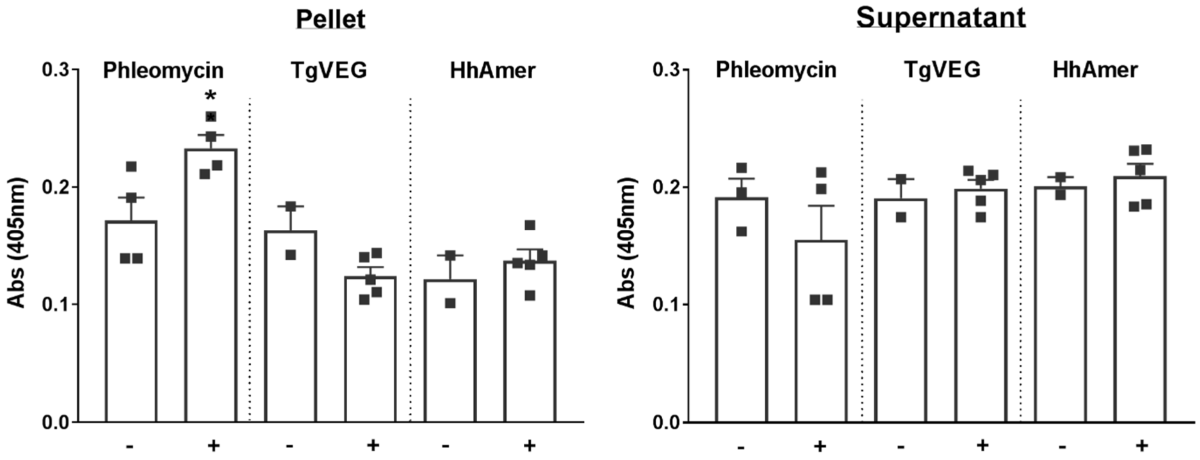

S5 Fig. β-galactosidase (gal) assay to detect activity of β-gal in THP-1 cells (Pellet) and supernatant . THP-1 cells were infected with *T. gondii* (TgVEG) or *H. hammondi* (HhAmer) sporozoites with an MOI of 1.6 for 72 h and cells and supernatants were collected to quantify β-gal activity. THP-1 cells were also treated with 50 μg/uL phleomycin for 72 h to induce senescence and β-gal secretion as a positive control. β-gal activity was significantly higher in the phleomycin-treated THP-1 cells as compared to untreated THP-1 cells, while neither TgVEG nor HhAmer infection significantly altered secreted or cell-associated β-gal activity (Tukey's multiple comparisons test, $p>0.05$).
