# Supplementary material for: Head-to-head comparisons of Toxoplasma gondii and its near relative Hammondia hammondi reveal dramatic differences in the host response and effectors with species-specific functions
Source: PLoS Pathog. 2020 Jun 23;16(6):e1008528. doi: 10.1371/journal.ppat.1008528 (PMC7360062; doi:10.1371/journal.ppat.1008528)
Supplement: S6 Fig — Genes shown were mean-centered and then hierarchically clustered. Genes shown are a subset of the “Fridman Senescence UP” gene set as described in the manuscript and arrowheads indicate key genes including cyclin-dependent kinases and DNA damage response genes such as members of the GADD45 family. These data suggest that prior infection with T. gondii suppresses the ability of H. hammondi to induce DNA damage response pathways in the host cell. (PDF) [file ppat.1008528.s006.pdf]

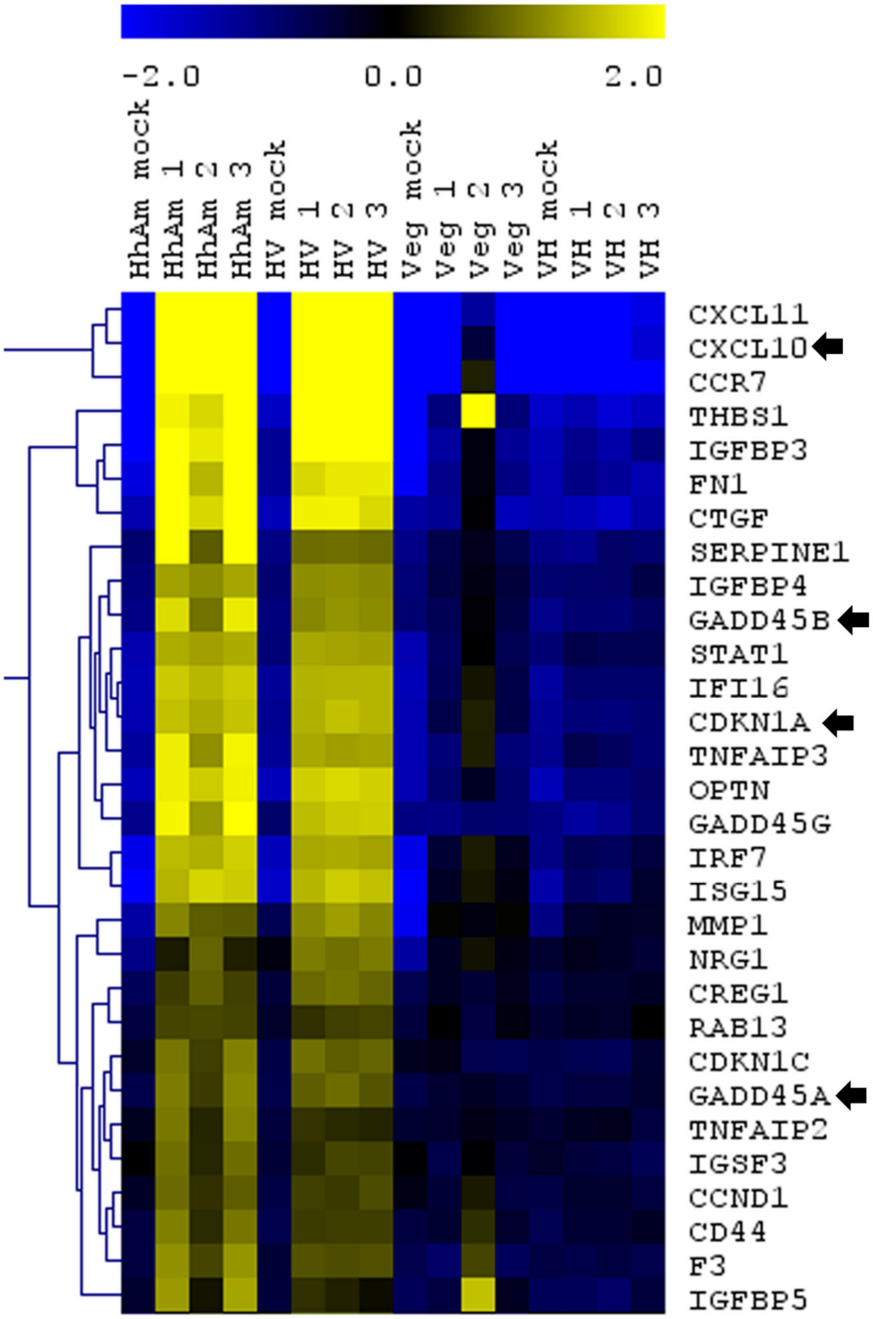

S6 Fig. Heatmap of $\log_2$(FPKM) RNAseq data from THP-1 cells infected with *H. hammondi*, *T. gondii* strain VEG, or pre-infection with one species followed by infection with the other (HV→*H. hammondi* and then *T. gondii*; VH→*T. gondii* VEG followed by *H. hammondi*). Genes shown were mean-centered and then hierarchically clustered. Genes shown are a subset of the "Fridman Senescence UP" gene set as described in the manuscript and arrowheads indicate key genes including cyclin-dependent kinases and DNA damage response genes such as members of the GADD45 family. These data suggest that prior infection with *T. gondii* suppresses the ability of *H. hammondi* to induce DNA damage response pathways in the host cell.
