# Supplementary material for: Head-to-head comparisons of Toxoplasma gondii and its near relative Hammondia hammondi reveal dramatic differences in the host response and effectors with species-specific functions
Source: PLoS Pathog. 2020 Jun 23;16(6):e1008528. doi: 10.1371/journal.ppat.1008528 (PMC7360062; doi:10.1371/journal.ppat.1008528)
Supplement: S7 Table — (DOCX) [file ppat.1008528.s013.docx]

| Organism | Gene | Primer sequences (5’ to 3’) | | References |
| --- | --- | --- | --- | --- |
|  |  | Forward | Reverse |  |
| Parasite | *T. gondii* GRA1 | TTAACGTGGAGGAGGTGATTG | TCCTCTACTGTTTCGCCTTTG | [34] |
|  | *H. hammondi* GRA1 | GAGGAGGTGATGGAGACTATGA | CTCTACTGTCTCGCCTTTGTTC | [34] |
| Mouse | Cxcl10 | GACGGTCCGCTGCAACTG | CTTCCCTATGGCCCTCATTCT | [86] |
|  | Ccl22 | GCAAGCCCTATTCTTCTGCC | AGGCATCATAGGATCAGGGC | NA |
|  | Gapdh | TGTGTCCGTCGTGGATCTGA | CCTGCTTCACCACCTTCTTGAT | [86] |
| Human | CXCL10 | TGATTTGCTGCCTTATCTTTCTGA | CAGCCTCTGTGTGGTCCATCCTTG | NA |
|  | CCL22 | GTGGTGTTGCTAACCTTC | GGCTCAGCTTATTGAGAATC | [24] |
|  | GAPDH | TGACGGTGCCATGGAATTTG | CACATCGCTCAGACACCATG | NA |

**Table S8. Primer sequences used for quantifying gene expression.**
